# A Copper–Zinc Cyanamide Solid-Solution Catalyst with Tailored Surface Electrostatic Potentials Promotes Asymmetric N-Intermediate Adsorption in Nitrite Electroreduction

**DOI:** 10.1021/jacs.5c00837

**Published:** 2025-02-18

**Authors:** Jiacheng Jayden Wang, Huong T. D. Bui, Xunlu Wang, Zhuoran Lv, Huashuai Hu, Shuyi Kong, Zhiqiang Wang, Lijia Liu, Wei Chen, Hui Bi, Minghui Yang, Tore Brinck, Jiacheng Wang, Fuqiang Huang

**Affiliations:** † The State Key Laboratory of High Performance Ceramics and Superfine Microstructure, Shanghai Institute of Ceramics, Chinese Academy of Sciences, Shanghai 200050, China; ‡ Center of Materials Science and Optoelectronics Engineering, University of Chinese Academy of Sciences, Beijing 100049, China; § Department of Chemistry, CBH, KTH Royal Institute of Technology, SE-100 44 Stockholm, Sweden; ∥ School of Environmental Science and Technology, Dalian University of Technology, Dalian 116024, China; ⊥ State Key Laboratory of Metal Matrix Composites, School of Materials Science and Engineering, Shanghai Jiao Tong University, Shanghai 200240, China; # Department of Chemistry, Western University, 1151 Richmond Street, London, ON N6A5B7, Canada; ∇ Department of Materials Design and Innovation, University at Buffalo, The State University of New York, Buffalo, New York 14260, United States; ○ Zhejiang Key Laboratory for Island Green Energy and New Materials, Institute of Electrochemistry, School of Materials Science and Engineering, Taizhou University, Taizhou 318000, China; ◆ Key Laboratory of Advanced Energy Materials Chemistry (Ministry of Education), Nankai University, Tianjin 300071, China

## Abstract

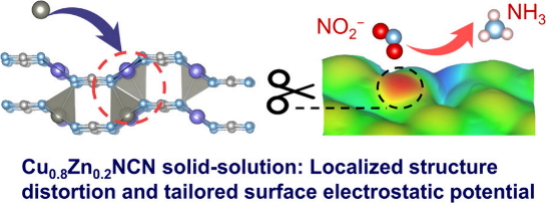

The electrocatalytic nitrite reduction (NO_2_RR) converts nitrogen-containing pollutants to high-value ammonia (NH_3_) under ambient conditions. However, its multiple intermediates and multielectron coupled proton transfer process lead to low activity and NH_3_ selectivity for the existing electrocatalysts. Herein, we synthesize a solid-solution copper–zinc cyanamide (Cu_0.8_Zn_0.2_NCN) with localized structure distortion and tailored surface electrostatic potential, allowing for the asymmetric binding of NO_2_^–^. It exhibits outstanding NO_2_RR performance with a Faradaic efficiency of ∼100% and an NH_3_ yield of 22 mg h^–1^ cm^–2^, among the best for such a process. Theoretical calculations and in situ spectroscopic measurements demonstrate that Cu–Zn sites coordinated with linear polarized [NCN]^2–^ could transform symmetric [Cu–O–N–O–Cu] in CuNCN-NO_2_^–^ to a [Cu–N–O–Zn] asymmetric configuration in Cu_0.8_Zn_0.2_NCN-NO_2_^–^, thus enhancing adsorption and bond cleavage. A paired electro-refinery with the Cu_0.8_Zn_0.2_NCN cathode reaches 2000 mA cm^–2^ at 2.36 V and remains fully operational at industrial-level 400 mA cm^–2^ for >140 h with a NH_3_ production rate of ∼30 mg_NH3_ h^–1^ cm^–2^. Our work opens a new avenue of tailoring surface electrostatic potentials using a solid-solution strategy for advanced electrocatalysis.

## Introduction

Ammonia (NH_3_) is a high-value chemical raw material for producing nitrogen-rich fertilizers and is also a promising energy carrier.^1−3^ Conventional NH_3_ production relies on the Haber-Bosch process, which operates under high temperature and pressure (∼500 °C, >100 atm),^4−6^ leading to significant carbon dioxide emissions and considerable energy consumption. Electrocatalytic nitrite reduction to ammonia (NO_2_RR) under ambient conditions offers significant kinetic advantages due to the low N=O bond dissociation energy (236 kJ mol^–1^).^7,8^ Additionally, nitrite (NO_2_^–^), derived from various industrial processes, is a major water pollutant, posing serious environmental challenges.^9,10^ Therefore, NO_2_RR offers a promising method for removing harmful NO_2_ and efficiently synthesizing ammonia.^11,12^ However, NO_2_RR involves a complex six-electron, seven-proton transfer process, and achieving optimal adsorption and balance of key intermediates, namely, NO_2_*, NO*, and H*, is crucial for minimizing the energy barrier of the rate-determining step (RDS).^13−17^ Hence, there is an urgent need to develop advanced electrocatalysts for the NO_2_RR with high activity and selectivity.

Recently, metal cyanamide compounds have emerged as a promising class of inorganic functional materials with significant application potential.^18−21^ Compared to O^2–^ inorganic anions, the long-chain conjugated cyanamide ion [NCN]^2–^ is a stronger electron acceptor, inducing more delocalized metal active sites and enhancing catalytic activity. Our research group designed and synthesized a new Cu_2_NCN material,^22^ where the [NCN]^2–^ anion promotes the transition of the original electronic structure of Cu sites into a more delocalized state, significantly improving the yield and selectivity of the CO_2_ reduction reaction (CO_2_RR) for methanol electro-synthesis. However, the application of metal cyanamide compounds in the NO_2_RR for NH_3_ synthesis remains unexplored. The nearly isolated metal active sites coordinated with long-chain [NCN]^2–^ anionic units in these compounds could exhibit unique properties in the NO_2_RR, particularly influencing the “six-electron seven-proton transfer process”.

It has been demonstrated that cost-effective copper-based materials can serve as efficient and robust NO_2_RR catalysts,^23−25^ primarily due to the partially filled d-orbitals of Cu, which facilitate the activation of NO_2_^–^. However, the multiorbital electrons in the d-block also favor the binding of H* (proton), promoting the competing hydrogen evolution reaction (HER) and reducing the selectivity of NO_2_RR and the yield of NH_3_. Zinc (Zn), which has lower electronegativity and fully occupied orbitals, exhibits a stronger oxygen affinity and weaker H-proton binding ability, thereby regulating the adsorption of NO_*x*_ intermediates and suppressing HER competition. This allows for better regulation of the adsorption configuration of nitrogen–oxygen intermediates, promoting a more efficient NO_2_RR conversion. On the other hand, solid-solution materials, which disperse foreign solute metal atoms within a metal solvent matrix, offer the combined advantages of enhanced active surface area and synergistic catalytic sites. These properties have attracted considerable attention especially in the catalytic process with a multielectron and multiproton process.

**Figure 1:**
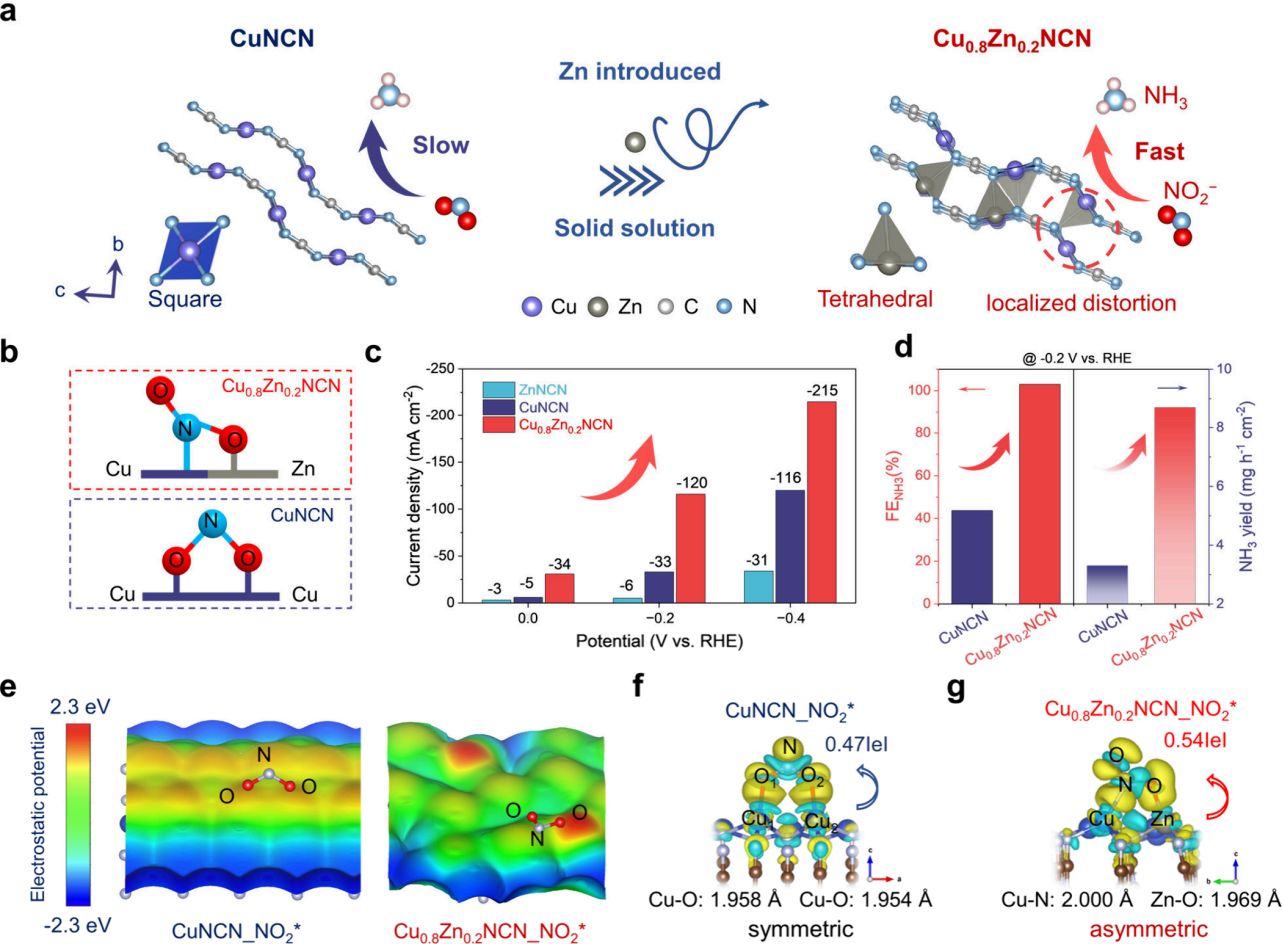
**Schematic illustration of designing Cu**_**0.8**_**Zn**_**0.2**_**NCN solid-solution electrocatalyst with asymmetric adsorption of NO**_**2**_^**–**^**to enhance NO**_**2**_**RR performance. a**, Schematic illustrating the synthetic strategy and structure of Cu_0.8_Zn_0.2_NCN solid-solution by introducing Zn into CuNCN. The Zn introduction leads to localized structure distortion. **b**, The asymmetric [Cu–N–O–Zn] and symmetric [Cu–O–N–O–Cu] configuration of NO_2_^–^ adsorption on Cu_0.8_Zn_0.2_NCN (upper) and CuNCN (lower). **c**, Current densities of Cu_0.8_Zn_0.2_NCN, CuNCN, and ZnNCN at 0, −0.2, −0.4 V vs RHE for NO_2_RR. **d**, NH_3_ Faradaic efficiency and NH_3_ yields for CuNCN and Cu_0.8_Zn_0.2_NCN at −0.2 V vs RHE. **e**, The surface electrostatic potential (*V*_S_(**r**)) at the 0.001 au isodensity surface of the bare CuNCN and Cu_0.8_Zn_0.2_NCN structures. **f, g**, Charge density difference, Cu–O/Cu–N, Zn–O bond length (Å), and adsorption energy for NO_2_* symmetric adsorption on CuNCN (**f**) and asymmetric adsorption on Cu_0.8_Zn_0.2_NCN (**g**). The isosurface value is set to 0.002 e/Å^3^. The yellow and cyan regions represent charge accumulation and depletion, respectively.

Herein, we explore a solid-solution cyanamide compound by incorporating Zn atoms into copper cyanamide, showing superior NO_2_RR activity and selectivity for ammonia synthesis. The solid solution of Zn has a locally distorted tetrahedral configuration of Cu_0.8_Zn_0.2_NCN, which effectively tailors the surface electrostatic potential (V_*S*_(**r**)) for NO_2_ binding and enhances the material stability (Figure 1a). Cu_0.8_Zn_0.2_NCN shows a Faradaic efficiency (FE) of ∼100% and an NH_3_ yield of 22 mg h^–1^ cm^–2^ at −0.5 V vs RHE. Theoretical calculations reveal the V_*S*_(**r**) of Cu_0.8_Zn_0.2_NCN is tailored for strong asymmetric binding of NO_2_^–^. A paired electro-refinery (PER) with Cu_0.8_Zn_0.2_NCN cathode demonstrates an impressive ammonia production rate of ∼30 mg h^–1^ cm^–2^ at an industrial-level current density of 400 mA cm^–2^, along with excellent stability for over 140 h of electrolysis. This work indicates the efficacy of constructing metal cyanamide compounds with tailored surface electrostatic potentials for advanced electrocatalytic processes.

## Results and Discussion

### Design of Cu_1–*x*_Zn_*x*_NCN Solid-Solution with Asymmetric Cu–Zn Sites and Tailored Surface Electrostatic Potentials

Our catalyst design of our group is based on linear polarized [NCN]^2–^ coordinated with Cu–Zn asymmetric sites (Figure 1b). The resulting Cu_0.8_Zn_0.2_NCN solid-solution exhibits a larger current density and higher FE_NH3_ values and NH_3_ yield rates than CuNCN with symmetric Cu sites for NO_2_RR (Figure 1c,d). This activity enhancement of NO_2_RR on Cu_0.8_Zn_0.2_NCN solid-solution over pristine CuNCN is further elucidated through the positive *V*_S_(**r**) and bonding nature.^26−28^ In CuNCN, highly positive surface maxima (*V*_S, max_) (1.49 eV) are found above surface Cu, attracting the negative O of NO_2_^–^. In Cu_0.8_Zn_0.2_NCN, *V*_S, max_ at Cu decreases to 0.93 eV, while Zn shows a much higher *V*_S, max_ of 2.27 eV due to its lower electronegativity (Figure 1e), thus adsorbing the O and N atoms of NO_2_^–^, respectively.

The electron density difference (EDD) was further carried out to investigate the surface intermediates’ adsorbed electronic states. Notably, NO_2_^–^ adsorbs symmetrically on CuNCN via two O–Cu bonds (Cu_1_–O_1_: 1.958 Å, and Cu_2_–O_2_: 1.954 Å) but adsorbs asymmetrically on Cu_0.8_Zn_0.2_NCN via one N–Cu (2.000 Å) and one O–Zn bond (1.969 Å) (Figure 1f,g). This asymmetric adsorption also leads to more electron transfer (0.54 e) in NO_2_*-adsorbed Cu_0.8_Zn_0.2_NCN than (0.47 e) in NO_2_*-adsorbed CuNCN. Therefore, the strong and asymmetric NO_2_* binding mode on the Cu_0.8_Zn_0.2_NCN surface drives N–O bond cleavage, which potentially improves the performance of the NO_2_RR.

### Structure Characterization of Cu_1–*x*_Zn_*x*_NCN Solid-Solution Electrocatalyst

**Figure 2:**
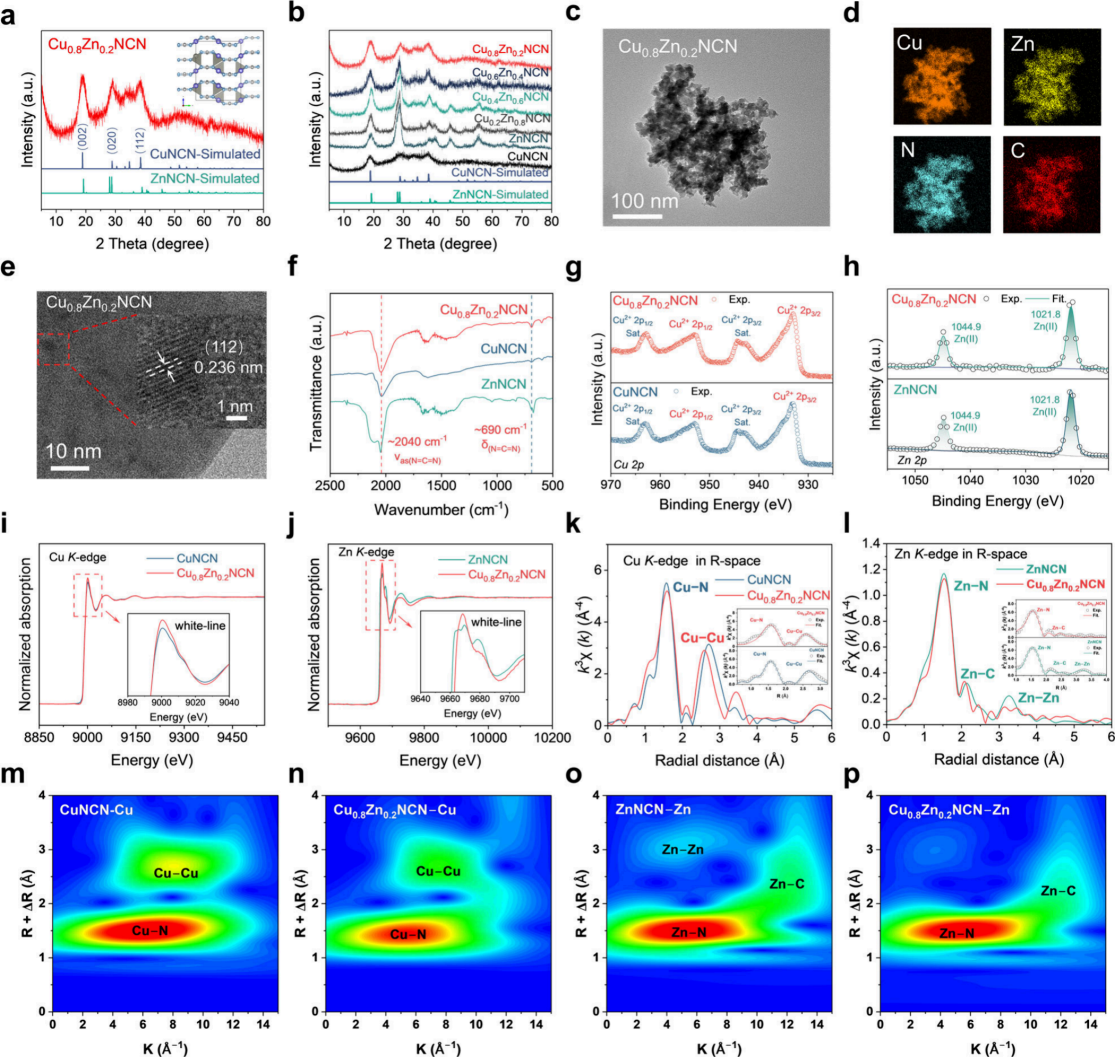
**Structure characterization of Cu**_**0.8**_**Zn**_**0.2**_**NCN solid-solution electrocatalyst. a**, XRD patterns of Cu_0.8_Zn_0.2_NCN and the corresponding CnNCN and ZnNCN (simulated). **b**, XRD patterns of Cu_1–*x*_Zn_*x*_NCN. **c-e**, High-angle annular dark-field scanning transmission electron microscopic (HAADF-STEM) image (**c**), energy-dispersive spectroscopic (EDS) mapping images (**d**), and HRTEM image (**e**) of Cu_0.8_Zn_0.2_NCN. **f**, FTIR spectra of Cu_0.8_Zn_0.2_NCN, CuNCN, and ZnNCN. **g, h**, High-resolution X-ray photoelectron spectroscopy (XPS) of Cu_0.8_Zn_0.2_NCN, CuNCN, and ZnNCN. Deconvoluted spectra of Cu 2p (**g**), Zn 2p (**h**). **i, j**, Normalized XANES spectra at the Cu K-edge of Cu_0.8_Zn_0.2_NCN and CuNCN (**i**) and Zn *K*-edge of Cu_0.8_Zn_0.2_NCN and ZnNCN (**j**). The illustration is an enlarged contrast of white line peaks. **k**, Corresponding Cu *K*-edge EXAFS fittings of Cu_0.8_Zn_0.2_NCN and CuNCN. **l**, Corresponding Zn *K*-edge EXAFS fittings of Cu_0.8_Zn_0.2_NCN and ZnNCN. **m-p**, Wavelet-transformed (W. T.) EXAFS patterns at Cu and Zn *K*-edge with the optimized Morlet parameter for Cu_0.8_Zn_0.2_NCN, CuNCN, and ZnNCN at the first coordination shell.

Cu_1–*x*_Zn_*x*_NCN solid-solution materials were synthesized with increasing Zn content (20, 40, 60, 80 mol %) in the precursor (Methods in the Supporting Information). The X-ray diffraction (XRD) patterns of the as-synthesized Cu_1–*x*_Zn_*x*_NCN exhibited characteristic peaks corresponding to both CuNCN and ZnNCN (Figure 2a). The XRD results indicate that CuNCN is the dominant phase when the Zn content is below 40 mol %, while ZnNCN becomes the dominant phase when the Zn content exceeds 60 mol % (Figure 2b). The Rietveld refinement was performed to accurately ascertain the crystal structures. The refined results unequivocally demonstrate that CuNCN adopts an Orthorhombic crystal system, whereas ZnNCN exhibits a Tetragonal crystal structure (Figure S1 and Table S1). And Cu_0.8_Zn_0.2_NCN has only one set of crystal structure at the Bragg site, and Zn replaces part of the Cu sites in it (Figure S2 and Table S2). Thermogravimetry analysis shows that the Zn introduction improves the thermal stability of the solid-solution materials (Figure S3). Furthermore, high-angle annular dark-field scanning transmission electron microscopy (HAADF-STEM) images and the corresponding energy dispersive X-ray spectroscopy (EDS) elemental mapping images indicate a homogeneous Zn distribution (Figure 2c,d). And the ratio of Cu/Zn is 4:1, which is consistent with the feed ratio (Figures S5 and S6 and Table S3), demonstrating the successful synthesis of a Cu_0.8_Zn_0.2_NCN solid-solution. The HR-TEM images show that the lattice spacing of 0.236 nm can be assigned to the Cu_0.8_Zn_0.2_NCN (112) plane (Figure 2e). Zn-free CuNCN exhibited a similar morphology and homogeneous Cu distribution (Figure S4). The Fourier transform infrared (FTIR) spectrum is shown in Figure 2f. The bending vibration around 690 cm^–1^ and the asymmetric stretching vibration around 2040 cm^–1^ imply the existence of NCN^2–^.^21^ In the Raman spectrum (Figure S7), the symmetric NCN^2–^ vibration is found as two peaks around 400 cm^–1^.^22^ The deconvoluted X-ray photoelectron spectroscopy (XPS) spectra indicate that Cu(II) and Zn(II) are the major surface species. The incorporation of Zn in CuNCN slightly alters the electronic structure of Cu, suggesting a possible interaction between Cu and Zn atoms (Figure 2g,h). Furthermore, the N 1s and C 1s spectra confirm that Cu and Zn bind to the N end of the [N=C=N]^2–^ anion and Cu_0.8_Zn_0.2_NCN shows the main CuNCN phase (Figure S8), consistent with the XRD result.

More detailed information about the Cu and Zn electronic states and local binding environments in Cu_0.8_Zn_0.2_NCN, CuNCN, and ZnNCN were deduced from the X-ray absorption near-edge structure (XANES) and the extended X-ray absorption fine structure (EXAFS). The XANES spectra white line peak of the Cu and Zn *K*-edge shape in Cu_0.8_Zn_0.2_NCN is closer to that of the Cu *K*-edge white line peak in CuNCN, while it is different from that of ZnNCN, proving that the chemical environment of Zn is similar to CuNCN (Figure 2i,j). The XANES spectra at N *K*-edge reveal that the intensity of the σ* band in Cu_0.8_Zn_0.2_NCN increased with respect to the π* band,^29^ demonstrating retention of C=N bonds and the increase of M-N coordination after Zn introduction, suggesting the stable states of Zn–N tetrahedra in Cu_0.8_Zn_0.2_NCN. For the comparison of Cu and Zn *K*-edge spectra (Figure S9), the energy absorption threshold of Cu_0.8_Zn_0.2_NCN is located near those of CuNCN and ZnNCN, suggesting that the valence state of Cu_0.8_Zn_0.2_NCN is situated at Cu^2+^ and Zn^2+^. Further fitting of the Fourier-transformed EXAFS spectra illustrate that Cu–N distances are around 1.981 Å (Cu_0.8_Zn_0.2_NCN) and 1.984 Å (CuNCN), and Zn–N are around 1.985 Å (Cu_0.8_Zn_0.2_NCN) and 1.977 Å (ZnNCN), respectively (Figure 2k,l). The Cu–N coordination number (CN) of Cu_0.8_Zn_0.2_NCN in the first shell is 4.8 (Table S4), substantially greater than that in CuNCN (CN = 4.0), revealing the localized distortion after Zn introduction. Zn in ZnNCN shows tetrahedral coordination, and the Zn–N coordination number (CN) is unchanged. Zn–Zn coordination is present only in ZnNCN, demonstrating the uniformly dispersed state of Zn in Cu_0.8_Zn_0.2_NCN. Moreover, the *k*-space information was revealed by the wavelet-transformed EXAFS spectra (Figures 2m–p and S10), in which Cu–N region (R_Cu–N_: ∼ 1.981 Å, k_Cu–N_: ∼ 6.5 Å^–1^) was found in the first coordination shell of Cu_0.8_Zn_0.2_NCN. The lower R_Cu–N_ value for Cu_0.8_Zn_0.2_NCN suggests a stronger Cu–N interatomic interaction compared to CuNCN (R_Cu–N_: ∼ 1.984 Å). The Zn–N coordination distance (R_Zn–N_: ∼ 1.985 Å), which is closer to R_Cu–N_ in CuNCN, and the fact that Zn–Zn is not coordinated both confirm the successful formation and uniform state of Cu_0.8_Zn_0.2_NCN. These results obtained from the EXAFS spectra are in agreement with the XPS and HR-TEM results, which further illustrate the solid-solution state of Cu_0.8_Zn_0.2_NCN.

### Electrocatalytic Performance of NO_2_RR on Cu_0.8_Zn_0.2_NCN Solid-Solution Electrocatalyst

**Figure 3:**
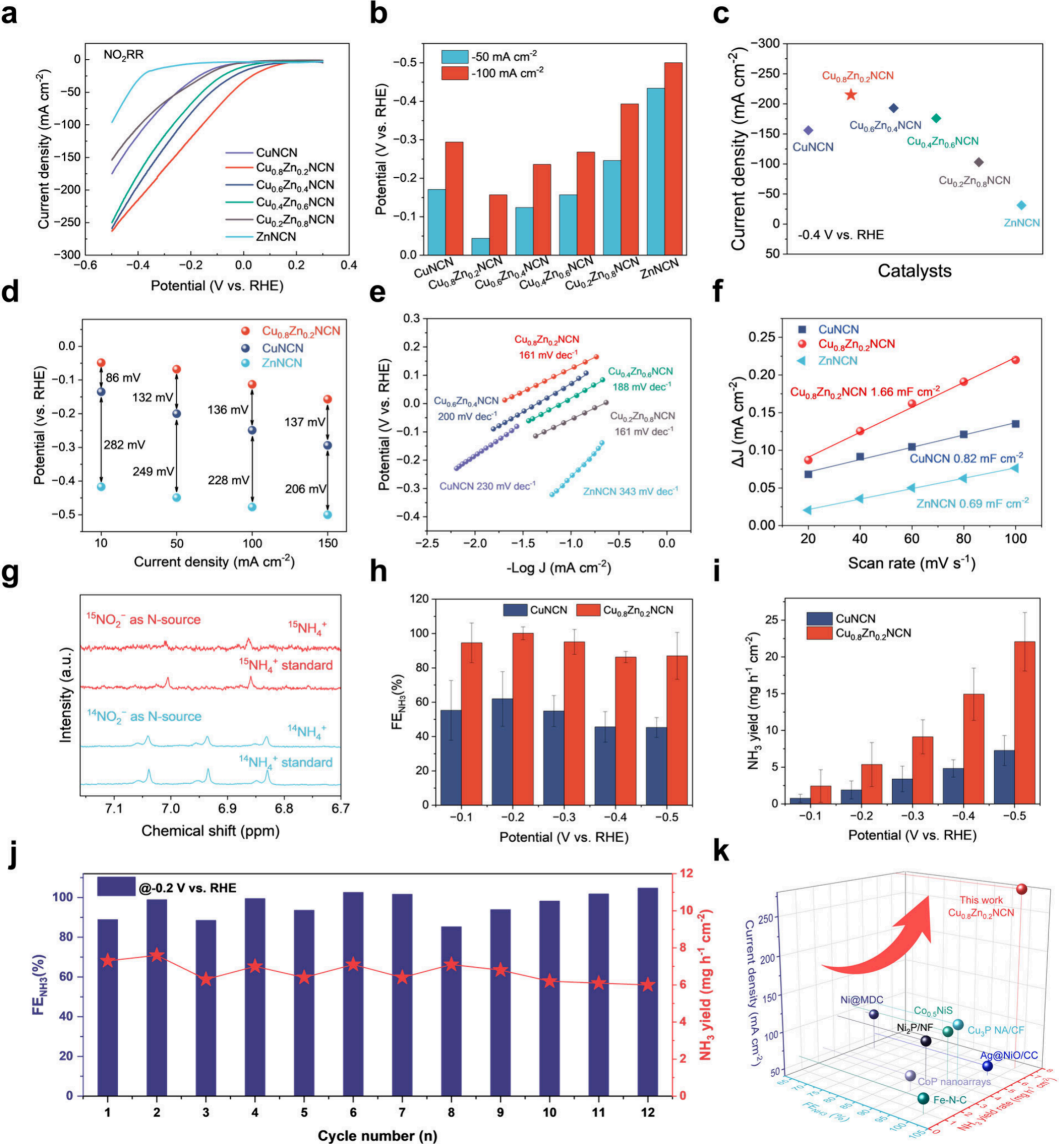
**Electrocatalytic performance of NO**_**2**_**RR by Cu**_**0.8**_**Zn**_**0.2**_**NCN solid-solution electrocatalyst and the control samples. a**, LSV curves of Cu_1–*x*_Zn_*x*_NCN. **b**, NO_2_RR overpotentials of CuNCN, Cu_1–*x*_Zn_*x*_NCN, and ZnNCN at −50 mA cm^–2^ and −100 mA cm^–2^. **c**, Current densities at −0.4 V vs RHE for Cu_1–*x*_Zn_*x*_NCN. **d, e**, Tafel plots (**d**) and Electrochemical Impedance Spectroscopy (**e**) of Cu_1–*x*_Zn_*x*_NCN. **f**, The calculated double-layer capacitance of Cu_0.8_Zn_0.2_NCN, CuNCN, and ZnNCN. **g**, NO_2_RR overpotentials of Cu_0.8_Zn_0.2_NCN, CuNCN, and ZnNCN vs RHE at different current densities. **h-i**, NH_3_ Faradaic efficiency (**h**) and NH_3_ yields (**i**) for CuNCN and Cu_0.8_Zn_0.2_NCN at the given potentials. **j**, The cyclic stability test and corresponding NH_3_ Faradaic efficiency and yields of Cu_0.8_Zn_0.2_NCN. **k**, The performance comparison of Cu_0.8_Zn_0.2_NCN solid-solution with the previously reported electrocatalysts toward NO_2_RR.

To determine the performance of NO_2_RR, the linear sweep voltammetry test of the Cu_0.8_Zn_0.2_NCN, CuNCN, and ZnNCN was first performed in 1 M KOH and 0.5 M KNO_2_ at 25 °C (Figure 3a and details in Supplementary Methods). For all samples with different Zn contents, Cu_0.8_Zn_0.2_NCN exhibits the lowest overpotential at 50 mA cm^–2^ and 100 mA cm^–2^ (Figures 3b, S11, and S12). Moreover, Cu_0.8_Zn_0.2_NCN also shows the highest current density compared to CuNCN, ZnNCN, and other Cu_1–*x*_Zn_*x*_NCN at −0.4 V vs RHE (Figure 3c). The Tafel slopes, calculated double-layer capacitance, and findings from Electrochemical Impedance Spectroscopy (EIS) all indicate a faster charge transfer rate for Cu_0.8_Zn_0.2_NCN (Figures 3d,e, S13, & S14).^30,31^ At all investigated current densities, Cu_0.8_Zn_0.2_NCN exhibits a lower overpotential than CuNCN and ZnNCN, suggesting a higher NO_2_RR activity of Cu_0.8_Zn_0.2_NCN (Figure 3f). The ^15^N isotope labeling experiments were conducted to further verify that the produced NH_3_ was resulting from the feeding NO_2_^–^ electrolyte.^32−34^ After electrolysis at −0.2 V versus RHE, triple coupling and doublet peaks corresponding to ^14^NH_4_^+^ and ^15^NH_4_^+^ were detected in the ^1^H NMR spectra of the electrolytes containing ^14^NO_2_^–^ and ^15^NO_2_^–^, respectively (Figure 3g). Cu_0.8_Zn_0.2_NCN shows higher Faradaic efficiency (FE) and ammonia yield at each potential than CuNCN. For the Cu_0.8_Zn_0.2_NCN sample, the maximum NH_3_ FE reaches ∼100% at −0.2 V versus RHE, and NH_3_ yield is as high as 22 mg h^–1^ cm^–2^ at −0.5 V versus RHE, both of which are significantly higher than those for CuNCN and ZnNCN (Figures 3h,i, S15, & S16).^35^ Meanwhile, in the case of NO_3_RR performance, a series of Cu_1–*x*_Zn_*x*_NCN have the same properties, but with lower NH_3_ selectivity than NO_2_RR (Figures S17–S19). The high performance of the Cu_0.8_Zn_0.2_NCN electrode was well-sustained during the cycling test (Figure 3j). After electrolysis, characterization by both powder XRD and electron microscopy indicated that Cu_0.8_Zn_0.2_NCN was preserved, with some degree of metallic state Cu formed (Figures S20 & S21). Such exceptional performance indicates that Cu_0.8_Zn_0.2_NCN solid-solution is highly competitive when compared to state-of-the-art transition-metal-based electrocatalysts used for NO_2_RR (Figures 3k & S22 and Table S5).

### NO_2_RR Mechanism Analysis of Cu_0.8_Zn_0.2_NCN Solid-Solution Electrocatalyst

**Figure 4:**
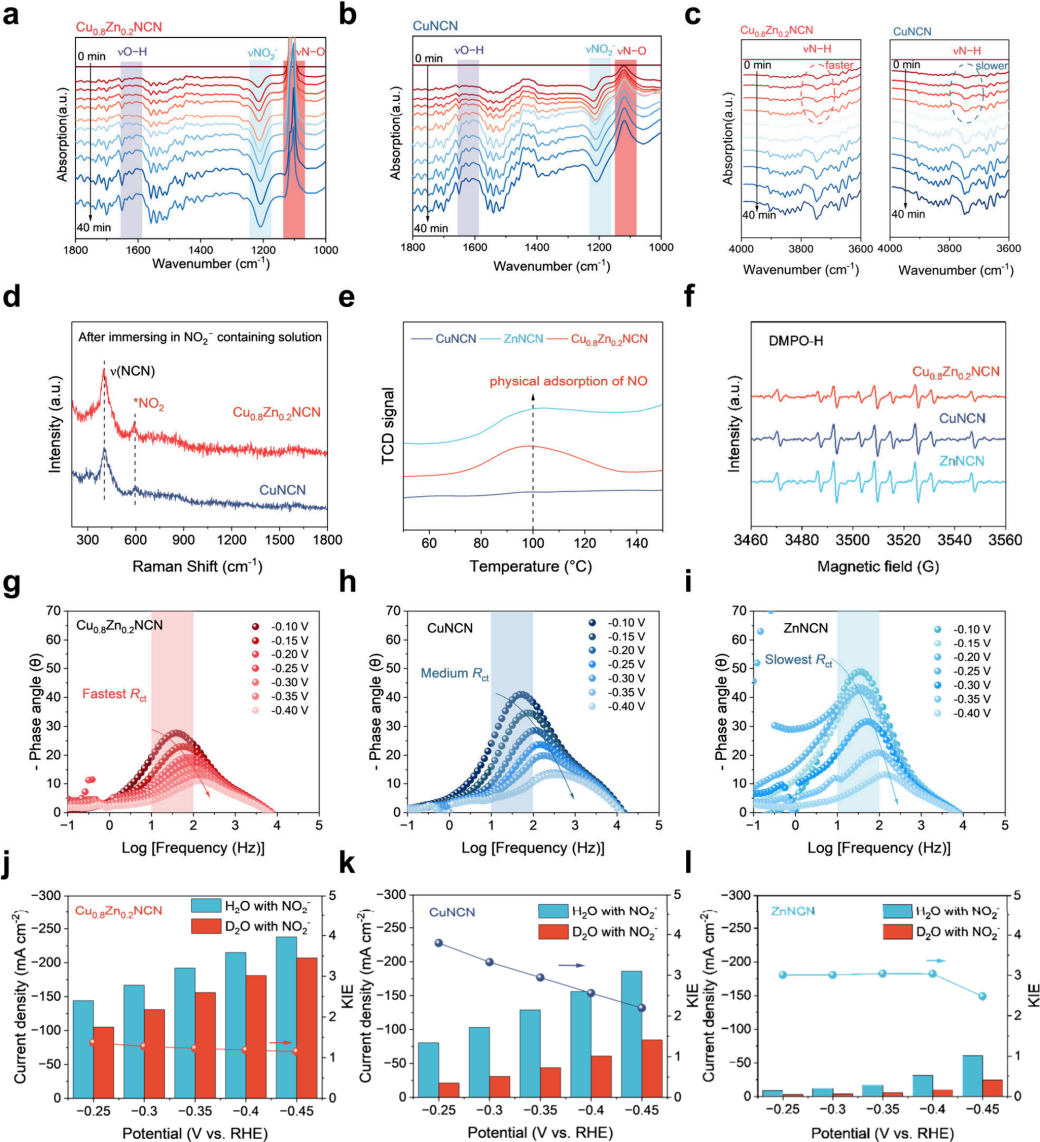
**NO**_**2**_**RR mechanism analysis of Cu**_**0.8**_**Zn**_**0.2**_**NCN solid-solution electrocatalyst. a-c**, In-situ FT-IR spectra of Cu_0.8_Zn_0.2_NCN and CuNCN under different reaction periods (0–40 min) of NO_2_RR at −0.1 V vs RHE. **d**, Raman spectra of Cu_0.8_Zn_0.2_NCN and CuNCN after immersing in NO_2_^–^ containing electrolyte for 24 h. **e**, NO adsorption curves of Cu_0.8_Zn_0.2_NCN, CuNCN, and ZnNCN were determined by TPD measurements. **f**, ESR spectra for Cu_0.8_Zn_0.2_NCN, CuNCN, and ZnNCN at −0.1 V (vs RHE) after 5 min of NO_2_RR in electrolyte containing DMPO. **g-i**, Bode phase plots of (g) Cu_0.8_Zn_0.2_NCN, (h) CuNCN, and (i) ZnNCN at different potentials. **j-l**, Calculated KIE values of Cu_0.8_Zn_0.2_NCN (j), CuNCN (k), and ZnNCN (l) catalysts from the LSVs in the H_2_O-based and D_2_O-based electrolyte containing 1.0 M KOH with 0.5 M KNO_2_ at various applied potentials.

The NO_2_RR mechanism of Cu_0.8_Zn_0.2_NCN was further investigated by various techniques. In-situ Fourier Transform Infrared (FT-IR) spectroscopy was performed to detect the intermediates and track the progress of the reaction. In the in situ FT-IR spectra of the Cu_0.8_Zn_0.2_NCN and CuNCN catalysts (Figure 4a,b), the positive band at 1100 cm^–1^ belongs to the *NO intermediates while the negative band at 1237 cm^–1^ can be attributed to the adsorption of *NO_2_.^36,37^ The positions obtained on Cu_0.8_Zn_0.2_NCN exhibit the clearer and stronger signals of *NO_2_ and *NO than CuNCN, further revealing the stronger adsorption of *NO, which is consistent with the better FE_NH3_ of Cu_0.8_Zn_0.2_NCN. Moreover, the emergence of obvious N–H stretching modes at 3730 cm^–1^ confirms the faster formation of NH_3_ on Cu_0.8_Zn_0.2_NCN than on CuNCN (Figure 4c). Raman spectra of Cu_0.8_Zn_0.2_NCN and CuNCN after immersing in NO_2_^–^ containing electrolyte were collected to investigate the adsorption ability of nitrogen oxides. The peak at ∼600 cm^–1^ can be classified as the symmetric NO_2_^–^ stretching associated with adsorbed NO_2_^–^, showing clearer and stronger signals at 600 cm^–1^ of NO_2_^–^ in Cu_0.8_Zn_0.2_NCN (Figures 4d, S23, & S24). This suggests Cu_0.8_Zn_0.2_NCN has enhanced adsorption of NO_2_^–^ compared to CuNCN. Temperature-Programmed Desorption (TPD) measurement shows that Cu_0.8_Zn_0.2_NCN exhibits the strongest adsorption capacity of NO in comparison to CuNCN and ZnNCN (Figures 4e and S25). In order to probe whether the H radicals (H*) existed during the NO_2_RR process, we monitored the formation of H* upon running NO_2_RR by Electron Paramagnetic Resonance (EPR) using dimethyl-1-pyrroline-*N*-oxide (DMPO) as the radical trapping reagent. Notably, in the presence of nitrite, the DMPO-H signals are stronger for CuNCN and ZnNCN, while those of Cu_0.8_Zn_0.2_NCN are the minimum occurrence (Figure 4f). This result indicates that the generated H* on the Cu_0.8_Zn_0.2_NCN surface participates in the hydrogenation of the activated nitrogen-containing intermediates on the adjacent surface and is rapidly consumed.

The reaction kinetics was probed by in situ EIS measurement. The Zn in Cu_0.8_Zn_0.2_NCN promotes electron and ion transfer during the NO_2_RR process. As is well-reported,the magnitude of the phase angle reflects the number of charges that participate in the Faraday process.^38^ The smaller the phase angle is, the more charges participate in the Faraday process. The Bode phase plots of the three samples manifest that, under the same bias, Cu_0.8_Zn_0.2_NCN exhibits a smaller phase angle than CuNCN and ZnNCN. This means that more charges in the Cu_0.8_Zn_0.2_NCN surface participate in the Faraday reaction instead of being stored in the electrode/electrolyte interface, while the opposite situation occurs for CuNCN and ZnNCN (Figures 4g–i).

To allow deeper insights into the kinetic behavior of surface active hydrogen species in the NO_2_RR under thermal field, the H/D kinetic isotope effect (KIE) investigation was performed to estimate the contribution of proton transfer in the rate-limiting step of electrocatalytic reactions.^39^ The LSV curves in KOH electrolytes with H_2_O and D_2_O as the solvent show apparent differences, while the KIE values for Cu_0.8_Zn_0.2_NCN that are calculated by the current density ratios (J_H2O_/J_D2O_) at specific potentials in Figure S26 are smaller compared to those of CuNCN and ZnNCN, indicating a faster hydrogen transfer kinetic rate during the HER. Notably, at all the applied potentials the KIE values for Cu_0.8_Zn_0.2_NCN are lower than those for CuNCN and ZnNCN, providing further evidence for the faster proton transfer rate during the NO_2_RR on Cu_0.8_Zn_0.2_NCN (Figure 4j–l).

### Theoretical Calculations on the NO_2_RR Mechanism

**Figure 5:**
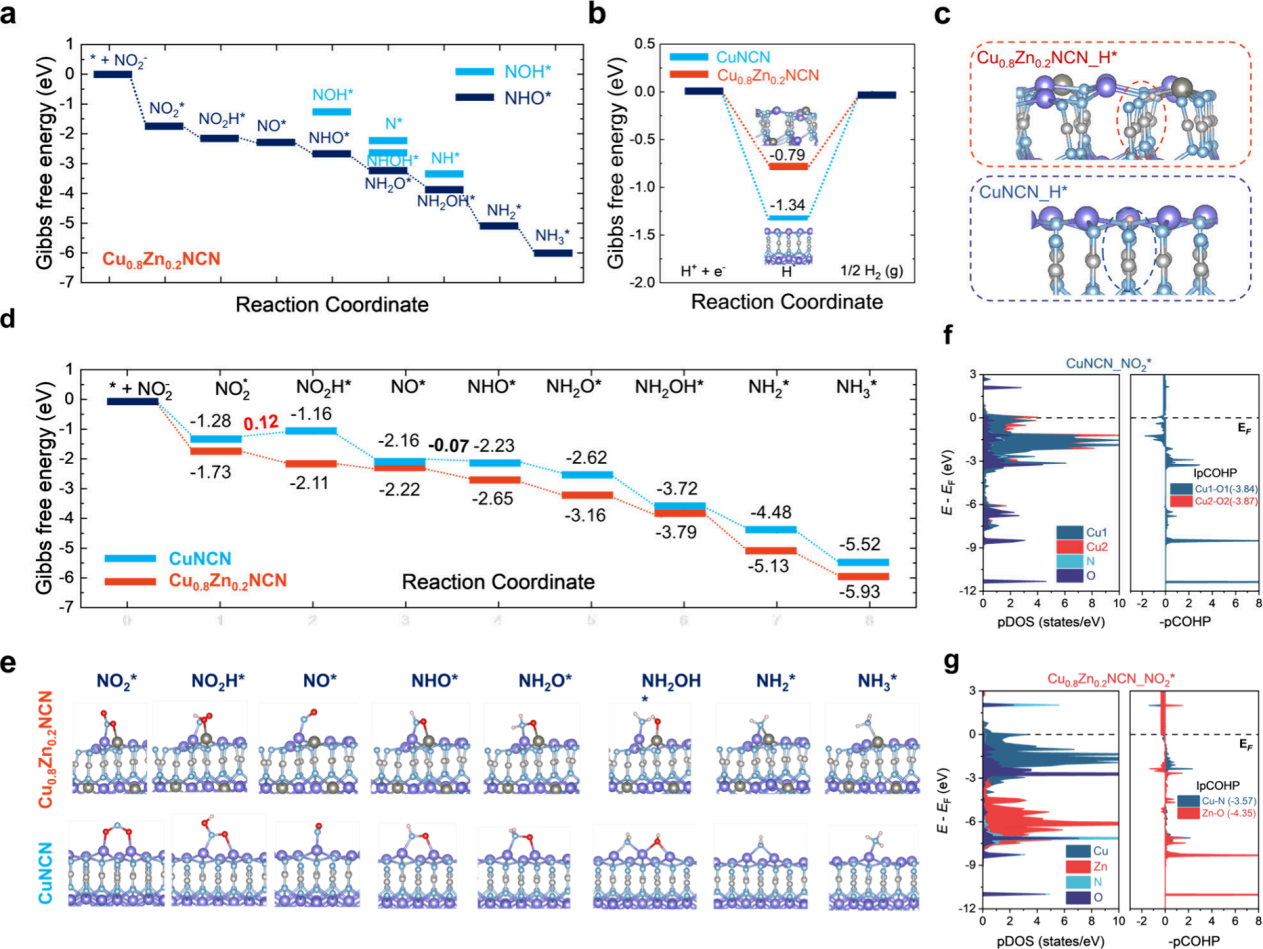
**Theoretical calculations on the NO**_**2**_**RR mechanism. a**, Free-energy diagrams for all possible reaction pathways of NO_2_^–^ reduction on Cu_0.8_Zn_0.2_NCN. **b**, Gibbs free energies of H* adsorption on CuNCN and Cu_0.8_Zn_0.2_NCN. **c**, The H* adsorption configurations on CuNCN and Cu_0.8_Zn_0.2_NCN. **d**, Free-energy profiles for the most thermodynamically favorable pathway of NO_2_^–^ reduction on CuNCN and Cu_0.8_Zn_0.2_NCN. **e**, The corresponding optimized intermediate geometries along all the NO_2_RR pathway on Cu_0.8_Zn_0.2_NCN (upper) and CuNCN (down). Color code: Cu-purple, N-light blue, C-gray, O-red, and H-pink. **f-g**, Partial density of states (PDOS) and projected crystal orbital Hamilton population (pCOHP) within the NO_2_* intermediate of CuNCN (**f**) and Cu_0.8_Zn_0.2_NCN (**g**) surface atoms.

To gain insight into the NO_2_RR mechanism, DFT calculations were conducted to investigate the NO_2_RR activity on Cu_0.8_Zn_0.2_NCN (002) (i.e., the strongest peak in XRD), in comparison with CuNCN (002). Considering the bimetallic effect on the electronic structure of cyanamide compound materials, we primarily investigated the electronic structure and performance of CuNCN and Cu_0.8_Zn_0.2_NCN models through DFT calculations and electrochemical tests (Figure S27 and Table S6). A comprehensive examination of the NO_2_RR performance via two possible reaction pathways, NHO* and NOH*, is conducted (Figures 5a and S28), showing the reaction energy change to create NHO* is downhill, while it is uphill for the formation of NOH*. The nitrite reduction preferentially proceeds via the NHO* pathway, generally known as the N-side pathway, with the sequence * + NO_2_^–^ → NO_2_* → NO_2_H* → NO* + H_2_O → NHO* → NH_2_O* → NH_2_OH* → NH_2_* + H_2_O → NH_3_*. In addition, Figure 5b,c shows that H* energetically prefers adsorption on N atoms of [NCN]^2–^ rather than on Cu or Zn atoms at both surfaces, implying that H* is not likely to block active sites during the NO_2_RR process. Notably, while the NO_2_RR on Cu_0.8_Zn_0.2_NCN proceeds without an energy barrier, the hydrogen evolution reaction (HER) requires 0.79 eV to proceed. The higher NO_2_RR selectivity over HER is also observed on CuNCN, where the energy barrier for the NO_2_RR is 0.12 eV, significantly lower than that for the HER of 1.34 eV. Thus, both surface structures favor the NO_2_RR over the HER. The overall lowest-energy reaction pathways with corresponding geometries of the NO_2_RR intermediates on Cu_0.8_Zn_0.2_NCN and CuNCN are shown in Figure 5d,e. One can see that the initial adsorption of NO_2_^–^ on both surfaces is a spontaneous process, with a greater energy release on Cu_0.8_Zn_0.2_NCN (−1.73 eV) than on CuNCN (−1.28 eV). Remarkably, the presence of Zn at the key active site, Cu–Zn, in Cu_0.8_Zn_0.2_NCN, as opposed to Cu–Cu in CuNCN, facilitates the favorable dissociation of the N–O bond during the first protonation step of NO_2_* to form stable NO–OH*. This results in a favorable Gibbs free energy change of −0.38 eV, compared to an energy demand of 0.12 eV for CuNCN. This step, NO_2_* → NO_2_H*, thus serves as the potential-determining step (PDS) for the entire NO_2_RR performance on CuNCN (Table S7).

After that, both structures feature an exergonic reduction step, forming NO* with the release of H_2_O. Despite an analogous tilt NO* adsorption mode via N–Cu, the Cu in Cu_0.8_Zn_0.2_NCN shows a slight preference for NO* adsorption over CuNCN, with free energy levels of −2.23 and −2.16 eV, respectively. Charge density difference and adsorption energy for NO* and NHO* adsorption on CuNCN and Cu_0.8_Zn_0.2_NCN and three above key species (NO_2_*, NO*, and NHO*) prefer to adsorb on Cu–Zn active sites (Cu_0.8_Zn_0.2_NCN): closely associated with more amount of charge acceptance, suggesting more effective charge transfer of the Cu_0.8_Zn_0.2_NCN catalyst compared to its counterpart (Figures S29 & S30).

Furthermore, more significant DOS overlaps between Cu and O in the NO_2_*-adsorbed CuNCN, lying in broader antibonding regions near the Fermi level (*E*_F_) (Figures 5f,g), reveal a weaker Cu–O bond strength compared to the Zn–O bond. This is quantitively verified by less negative IpCOHP values (−3.86 on average versus −4.35). The asymmetric NO_2_* binding mode on the Cu_0.8_Zn_0.2_NCN surface drives the N–O bond breakage under further proton attack, which is not observed on the CuNCN. In other words, the NO_2_* on Cu_0.8_Zn_0.2_NCN is favorable for the formation of NO–OH* intermediates, whereas a large geometric rearrangement is needed to form the NO_2_H* on CuNCN. The role of the favorable Zn–O bond is reinforced in the stable formation of the NHO* intermediate during the third protonation of the NO* step, attributed to more populated binding states of the Zn–O bond (highlighted in red) across the *E*_F_ compared to the Cu–O bond, accompanied by more negative IpCOHP values (−3.97 and −3.80, respectively) (Figures S31 & S32).

### Continuous Production of Ammonia in MEA Reactor

**Figure 6:**
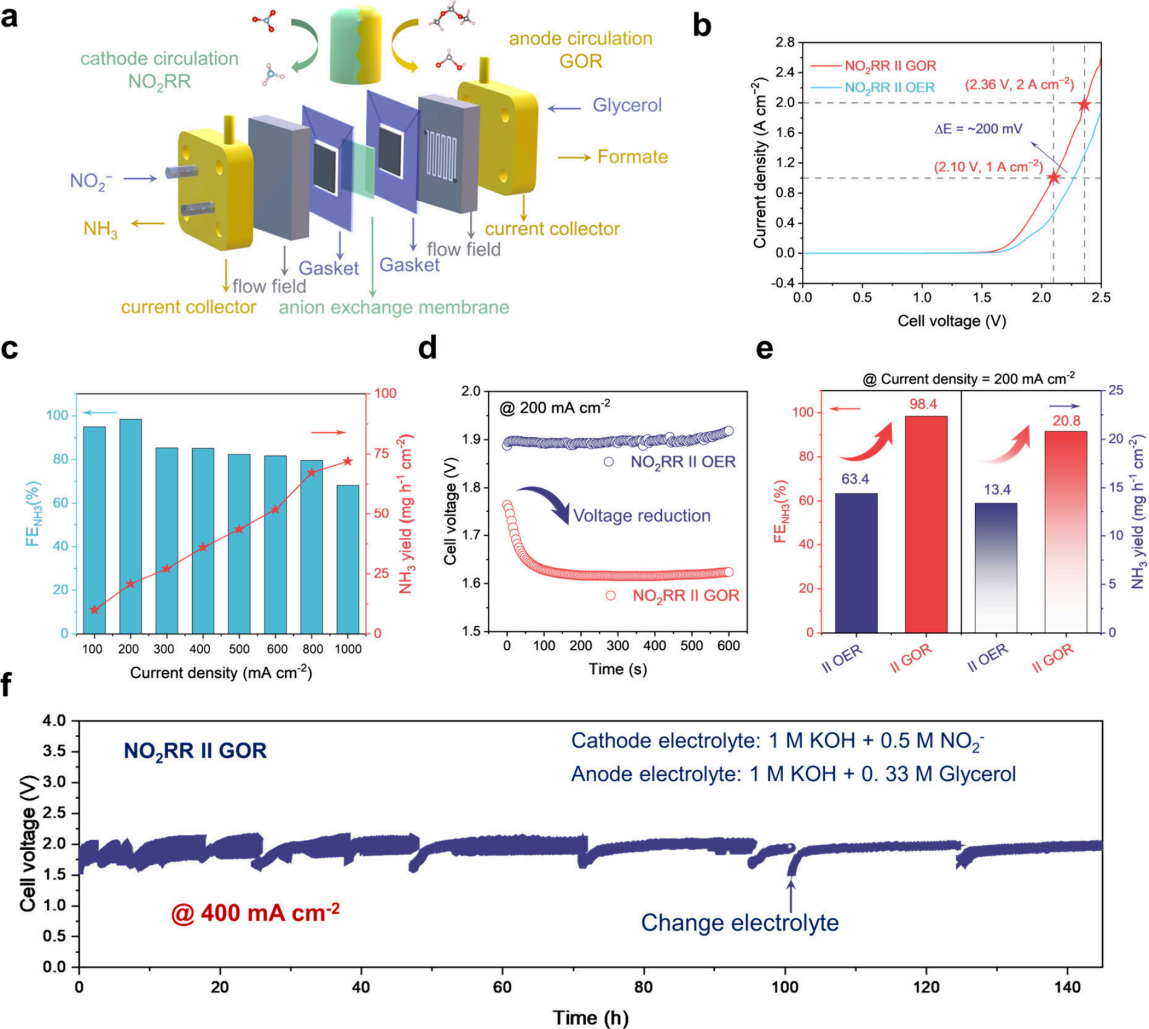
**Continuous coproduction of ammonia and formate in the PER system using MEA reactor. a**, Schematic of the PER system coupling NO_2_RR and GOR for green NH_3_ and formate electrosynthesis. **b**, V–I curves of the PER system with and without glycerol at the anode. **c**, Ammonia yield rate and FE_NH3_ in the PER system. **d**, Cell voltage versus operation time of NO_2_RR **||** GOR and NO_2_RR **||** OER system. **e**, Comparison of FE_NH3_ and ammonia yield rate at 200 mA cm^–2^ between **||** the OER and **||** the GOR. **f**, Continuous tests of the Cu_0.8_Zn_0.2_NCN solid-solution catalyst for over 140 h in the PER system using an MEA-based electrolyzer.

To investigate practical applications of solid-solution Cu_0.8_Zn_0.2_NCN catalyst for ammonia synthesis, we constructed a paired electro-refinery (PER) by coupling cathodic NO_2_RR and anodic glycerol oxidation reaction (GOR) using a membrane-electrode assembly (MEA)-based electrolyzer (Figure 6a).^40,41^ After changing the electrolyte of the anode compartment from pure KOH to KOH with glycerol, our paired electrorefinery voltage dropped by ∼200 mV at a current density of 1000 mA cm^–2^, and it could reach 2000 mA cm^–2^ at 2.36 V (Figure 6b).^42−44^ Besides, the NH_3_ yield rates and FE under industrial current densities are investigated (Figure 6c). The constructed PER system enables the ampere-level current density, where the NH_3_ yield rate could reach up to 73 mg_NH3_ h^–1^ cm^–2^ and the FE could reach 80.0% under 800 mA cm^–2^ (Figure S33 and Table S8). As shown in Figure 6d, this PER reactor (NO_2_RR **||** GOR) requires a significantly lower voltage relative to that of the NO_2_RR **||** OER system, achieving 200 mA cm^–2^ under ∼1.6 V, which has higher FE and NH_3_ yield rates (Figure 6e). Moreover, the coupled MEA remained stable at industrial-level 400 mA cm^–2^ for over 140 h with a NH_3_ production rate of ∼30 mg_NH3_ h^–1^ cm^–2^ (Figure 6f). This result indicates the industrial potential of the paired valorization route with polarized Cu_0.8_Zn_0.2_NCN for the NO_2_RR coupled system.

## Conclusions

In summary, our study highlights the Cu_0.8_Zn_0.2_NCN cyanamide solid-solution as an excellent NO_2_RR electrocatalyst for ammonia production. The introduction of Zn into CuNCN not only improves the stability of the material but also tailors the surface electrostatic potential for the NO_2_RR. Through a strategic combination of in situ electrochemical experiments and DFT calculations, we elucidate that the exceptional NO_2_RR ability of Cu_0.8_Zn_0.2_NCN can be ascribed to asymmetric adsorption of NO_2_^–^, promoting efficient N–O bond cleavage and enhancing ammonia selectivity. The Cu_0.8_Zn_0.2_NCN solid-solution exhibits a Faradaic efficiency (FE) of ∼100% and a maximum NH_3_ yield of 22 mg h^–1^ cm^–2^ at −0.5 V vs RHE. Moreover, the as-constructed PER system with Cu_0.8_Zn_0.2_NCN as cathode consistently achieves highly efficient production of ammonia at an industrial-level current density of 400 mA cm^–2^ for over 140 operational hours. This work presents a new idea into efficient NO_2_^–^-to-NH_3_ conversion by solid-solution bimetal cyanamide electrocatalysts with tailored surface electrostatic potentials. It is believed that this strategy can be broadly applied to other catalytic systems for synthesizing higher-value products.

## Supporting Information Available

Detailed experimental procedures, synthesis, characterization methods, and additional of characterization the materials (PDF)
