# Supplementary material for: A Copper–Zinc Cyanamide Solid-Solution Catalyst with Tailored Surface Electrostatic Potentials Promotes Asymmetric N-Intermediate Adsorption in Nitrite Electroreduction
Source: J Am Chem Soc. 2025 Feb 18;147(9):8012–23. doi: 10.1021/jacs.5c00837 (PMC11887442; doi:10.1021/jacs.5c00837)
Supplement: Supplementary file 1 — ja5c00837_si_001.pdf [file ja5c00837_si_001.pdf]

Supplementary Information

A Copper-Zinc Cyanamide Solid-Solution Catalyst with Tailored Surface Electrostatic Potentials Promotes Asymmetric N-intermediate Adsorption in Nitrite Electro-reduction

Jiacheng (Jayden) Wang[1,2], Huong T. D. Bui[3], Xunlu Wang[4], Zhuoran Lv[5], Huashuai Hu[4], Shuyi Kong[5], Zhiqiang Wang[6], Lijia Liu[6], Wei Chen[7], Hui Bi[1], Minghui Yang[4,*], Tore Brinck[3,*], Jiacheng Wang[1,8,9*] and Fuqiang Huang[1,5,*]

[1] The State Key Laboratory of High Performance Ceramics and Superfine Microstructure, Shanghai Institute of Ceramics, Chinese Academy of Sciences, Shanghai 200050, China

[2] Center of Materials Science and Optoelectronics Engineering, University of Chinese Academy of Sciences, Beijing 100049, China

[3] Department of Chemistry, CBH, KTH Royal Institute of Technology, SE-100 44 Stockholm, Sweden

[4] School of Environmental Science and Technology, Dalian University of Technology, Dalian 116024, China

[5] State Key Laboratory of Metal Matrix Composites, School of Materials Science and Engineering, Shanghai Jiao Tong University, Shanghai 200240, China

[6] Department of Chemistry, Western University, 1151 Richmond Street, London, ON N6A5B7, Canada

[7] Department of Materials Design and Innovation, University at Buffalo, The State University of New York, Buffalo, NY 14260, USA

[8] Zhejiang Provincial Key Laboratory for Island Green Energy and New Materials, Institute of Electrochemistry, School of Materials Science and Engineering, Taizhou University, Taizhou 318000, China

[9] Key Laboratory of Advanced Energy Materials Chemistry (Ministry of Education), Nankai University, Tianjin 300071, China

Corresponding authors: huangfq@sjtu.edu.cn (F. Huang); jiacheng.wang@tzc.edu.cn (J. Wang); tore@kth.se (T. Brinck); myang@dlut.edu.cn (M. Yang)

Table of Contents:

- Supplementary Methods
- Supplementary Figures S1–33
- Supplementary Tables S1–8

Supplementary Methods

Synthesis of $Cu_{1-x}Zn_xNCN$ solid-solution. Copper chloride (2.5(1-x) mmol) and zinc chloride (2.5x mmol) were first dissolved in deionized water (50 mL) at room temperature, followed by adding NaOH (8 mmol) and H_2NCN (5 mmol). Finally, the product was obtained by vacuum freeze-drying after filtration and washed with deionized water. If x = 0, the final product CuNCN was obtained. If x = 1, pure ZnNCN was formed.

Electrochemical measurements

Electrochemical measurements were performed in a standard three-electrode system (H-type electrolytic cell, Jiangsu BOKE New Materials Technology Co., LTD.) using a CHI 760E electrochemical workstation. In such a system, for alkaline electrolytes (i.e., 1.0 M KOH aqueous solution with or without 0.5 M NO_2^-), a $Hg/HgCl_2$ electrode and a graphite rod were used as the reference electrode and counter electrode, respectively. The catalyst ink was prepared by dispersing 10 mg catalyst ($Cu_{1-x}Zn_xNCN$) and 2 mg Super P carbon black in 1000 μl ethanol containing 50 μl of 5 wt% Nafion, followed by sonication for 45 min to generate the catalyst ink. The as-prepared electrodes were used as the working electrode with catalyst loading of 2 mg cm^{-2}. Fumasep FAB-PK-130 was used as an anion exchange membrane (AEM), provided by Jiangsu BOKE Co., LTD. All the potentials vs. $Hg/HgCl_2$ were converted to the values versus reversible hydrogen electrode (RHE) according to the equation (E vs. RHE = E vs. $Hg/HgCl_2$ +0.0592 × pH + 0.244 V). Linear sweep voltammetry (LSV) polarization curves were recorded at a scan rate of 5 mV s^{-1}. For the catalytic potential, we did not use iR correction. Before the LSV test, we conducted ten cycles of cyclic voltammetry measurements (100 mV s^{-1}) to clean the catalyst surface. Unless otherwise stated, all linear voltammetry curves were recorded after three prescans to achieve stabilization. EIS tests were conducted from 0.1 Hz to 1 MHz, and the results are presented in the form of a Bode phase plot.

Membrane Electrode Assembly (MEA) tests in a paired electro-refinery

For testing in a flow electrolyzer (Model: BKT2-SN-22-8X, manufactured by Jiangsu BOKE New Materials & Technology Co., Ltd., China), we prepared a membrane electrode assembly (MEA) by sandwiching the $Cu_{0.8}Zn_{0.2}NCN$ cathode and Ni foam anode between a commercial membrane (Fumasep FAB-PK-130). The MEA was then placed within a custom-designed electrolyzer where 1 M KOH with 0.5 M KNO_2 as the catholyte and 1 M KOH with 0.33 M glycerol as the anolyte was circulated through cathode and anode, respectively at a flow rate of 60 mL min^{-1}.

TPD for NO adsorption

All TPD studies were conducted in a quartz reactor. Typically, 50 mg of adsorbent was loaded into the reactor. A quartz wool plug was placed below the bed to prevent the adsorbent from entering the effluent gas line. Before NO adsorption measurements, the adsorbent underwent hydrothermal aging (HTA) in a stream of air containing 5% water from 348 K to 773 K at 2 K/min. The temperature was held at 773 K for 5 h, and then cooled back to 348 K in the absence of water vapor.

In-situ Raman analyses

In-situ Raman spectra were recorded on a micro-Raman spectrometer (Renishaw) under an excitation of 532 nm laser light under controlled potentials by the CHI 630E electrochemical workstation. $Cu_{0.8}Zn_{0.2}NCN$ deposited on glassy carbon was used as a working electrode. A Pt wire as the counter electrode was rolled to a circle around the cell. Ag/AgCl electrode (sat. KCl) was used as the reference electrode. The in-situ Raman spectra were collected under chronoamperometry (I-t) at different potentials in 1 M KOH with 0.5 M KNO_2 solution.

In-situ IR analyses

The in-situ infrared spectroscopy measurements were carried out on a Nicolet 6700 FT-IR spectrometer (Thermo Scientific, USA) equipped with a liquid-nitrogen cooled MCT-A

detector. For continuously collection of in-situ FT-IR spectra during NO_2RR, the catalyst (10 μg) was supported on a glassy carbon electrode, which served as the working electrode. A saturated Hg/HgO electrode and a Pt wire were used as the reference and counter electrode, respectively. The electrochemical cell was filled with the specific reaction solution. Each spectrum was scanned 32 times with a spectral resolution of 4 cm^{-1} and a time interval of 0.2 min during the electrochemical reduction process.

XANES and EXAFS analyses

The Cu K-edge XAFS were measured at the SXRMB beamline at the Canadian Light Source. The spectra were recorded in fluorescence mode with normalization to the incident photon flux. All spectra were processed using the ATHENA software (version 0.9.26.10). EXAFS fitting was performed with FEFF models within an R range of 1 Å and 2 Å (first shell only). The spectra were obtained through Fourier transform at k-range of 3 $Å^{-1}$ and 11.7 $Å^{-1}$. The wavelet transformation (W. T.) of $\chi(k)$ was conducted on a python-based signal.

Detection of NH_4^+

The quantification of NH_4^+ was conducted using the indophenol blue method. To mitigate experimental errors associated with ammonia quantification, all data were recorded in triplicate to generate error bars. Then, 2 mL of a 1 M sodium hydroxide solution containing 5% salicylic acid and 5% sodium citrate was introduced. Subsequently, 1 mL of 0.05 M sodium hypochlorite and 0.2 mL of 1 wt% $C_5FeN_6Na_2O$ were added to the collected electrolyte solution. After being left standing for 30 min, the absorbance at 656 nm was measured by UV-spectroscopy. The obtained value was then fitted to the calibration curve to acquire the corresponding NH_4^+ concentration. The concentration-absorbance curve was calibrated using a series of standard ammonium chloride solutions (0.1, 0.2, 0.5, 1, 2, 3 mg L^{-1} NH_4^+-N), and the ammonium chloride crystal was dried at ~105−110 °C for 2 h in advance.

Calculation of NH_3 Faradaic efficiency and yield rate.

The NH_3 Faradaic efficiency was calculated according to the following equation:

$$FE_{NH3} = \frac{Q_{NH3}}{Q} = \frac{n_{NH3}V_{NH3}F}{Q}$$

where Q represents the applied overall coulomb quantity (C), Q_{NH3} is the coulomb required to produce NH_3, n is the electron-transfer number (for 1 mol NH_3, it is 6), V is the volume of the catholyte of the cathode chamber (30 ml), C_{NH3} is the concentration of NH_3 produced, and F is the Faraday constant (96,485 C mol^{-1}).

The NH_3 yield rate is calculated by the following equation:

$$NH_3\, yield = \frac{c_{NH3} \times V}{t \times A}$$

where c_{NH3} is the produced NH_3 concentration, V is the volume of the cathode electrolyte solution in L, t is the reduction time in h, A is the area of the carbon paper in cm^2.

Isotope labeling experiments.

Isotope labeling experiments were conducted using $Na^{15}NO_2$ as the N source to investigate the source of NH_3. The electrolyte containing $^{15}NH_4^+$ was taken and the pH of the solution was adjusted to 2 by adding 1 mL of 3 M H_2SO_4. Subsequently, 0.45 mL of mixed solution, and 50 μL of maleic acid solution (0.1 g maleic acid dissolved in 25 mL D_2O) were transferred to the NMR tube.

The glycerol oxidation products analysis

The glycerol oxidation products were determined by high performance liquid chromatography (HPLC). The electrolysis reactions of glycerol oxidation were carried out at different current densities by chronoamperometry in 50 mL electrolyte of 1 M KOH with 0.33 M glycerol. 5.0 mL electrolyte was extracted after glycerol electrooxidation, and then was diluted with H_2SO_4 solution to adjust the pH below 7.0. 20 μL diluted solution was injected into the column. 0.27 mL H_2SO_4 diluted in 1000 mL H_2O was used as eluent with a constant flow rate of 0.6 mL/min. All the electrolysis experiments were carried out at room temperature and all the HPLC

measurements were performed with a column temperature of 60 ºC. The composition of electrolyte after glycerol oxidation was identified based on the retention times of HPLC elution peaks of the individual standard sample. The product concentration was determined by the calibration curves of standard solutions with given concentrations.

The product selectivity ($S_{glycerate}$, $S_{glycolate}$, and $S_{formate}$, respectively), faradaic efficiency, and glycerol conversion ($\eta_{glycerol}$) were calculated by the following equations:

The product selectivity ($S_{glycerate}$, $S_{glycolate}$, and $S_{formate}$, respectively) is calculated by the following equation:

$$Selectivity\ (\%) = \frac{concentration\ of\ the\ product}{concentration\ of\ all\ products} \times 100\%$$

The Faradaic efficiency calculations of the glycerol oxidation production are based on the following balance half-reactions:

$C_3H_8O_3$ (glycerol) + 5 OH^- → $C_3H_5O_4^-$ (glycerate) + 4 H_2O + 4 e^-

$C_3H_8O_3$ (glycerol) + 13/2 OH^- → 3/2 $C_2H_3O_3^-$ (glycolate) + 5 H_2O + 5 e^-

$C_3H_8O_3$ (glycerol) + 11 OH^- → 3 $HCOO^-$ (formate) + 8 H_2O + 8 e^-

The corresponding Faradaic efficiencies (FE) toward glycerate, glycolate, and formate are calculated based on the following equations:

$$FE_{glycerate} = \frac{C_{glycerate} \times 4}{Q_{total}} \times V \times F \times 100\%$$

$$FE_{glycolate} = \frac{C_{glycolate} \times \frac{2}{3} \times 5}{Q_{total}} \times V \times F \times 100\%$$

$$FE_{formate} = \frac{C_{formate} \times \frac{1}{3} \times 8}{Q_{total}} \times V \times F \times 100\%$$

where $C_{glycerate}$, $C_{glycolate}$, and $C_{formate}$ are the final concentrations of glycerate, glycolate, and formate, respectively; V is the volume of the electrolyte solution; F is the

Faraday's constant (96485 C/mol); Q_{total} is the charge passed.

The glycerol conversion ($\eta_{glycerol}$) is calculated by the following equation:

$$\eta_{glycerol} = \frac{C_{0,glycerol} - C_{glycerol}}{C_{0,glycerol}} \times 100\%$$

where the $C_{0,glycerol}$ and $C_{glycerol}$ are the initial and final concentration of glycerol, respectively.

Computational Details

DFT calculations were implemented using Vienna ab initio simulation package (VASP)[1] using the Perdew, Burke and Ernzerhof (PBE) functional[2] within the generalized gradient approximation (GGA)[3]. A plane wave cutoff of 450 eV was employed. For model structures, the 5x2 supercell of CuNCN(200) surface was created, consisting of 40 Cu, 40 C and 80N atoms. For the $Cu_{0.8}Zn_{0.2}NCN$, 8 Cu atoms were substituted with 8 Zn atoms within the CuNCN and the substitution pattern that gave the lowest energy of the bulk structure after structure optimization was selected (shown in **Figure S27**). A 15 Å vacuum space length along the *z* direction was set to minimize interlayer interactions. The Brillouin zone was sampled by using Monkhorst–Pack with 2x3x1 and 3x4x1 *k*-point sampling for geometric optimization which was selected (shown in **Table S5**) and electronic property calculations, respectively. The convergence tolerance of energy and force was taken to be 0.02 eV/Å and 10^{-5} eV, respectively. The DFT-D3 method of Grimme was used to calculate dispersion corrections.[4] The surface electrostatic potential at the 0.001 au electron isodensity contour was computed from the CHGCAR and LOCPOT files.[5] VESTA was used for the surface electrostatic potential plots.[6] VASPKIT was used for data-postprocessing of PDOS.[7] The projected Crystal Orbital Hamilton Populations (pCOHP) calculations and Mulliken charge analyses were performed by the LOBSTER package.[8]

According to the computational hydrogen electrode (CHE) model,[9] the Gibbs free-energy

change (ΔG) was calculated as $\Delta G = \Delta E + \Delta E_{ZPE} - T\Delta S$ where ΔE, $\Delta E_{ZPE,}$ and ΔS are the total energy difference, zero-point energy change and entropy change , with temperature (T) was set to 298.15 K. Limiting potential (U_L) was defined via the equation: $U_L = -\Delta G_{max}$/ne, where ΔG_{max} is the free energy difference of the potential determining step (PDS) and ne is number of electrons transferred in the elementary reaction step.

Supplementary Figures

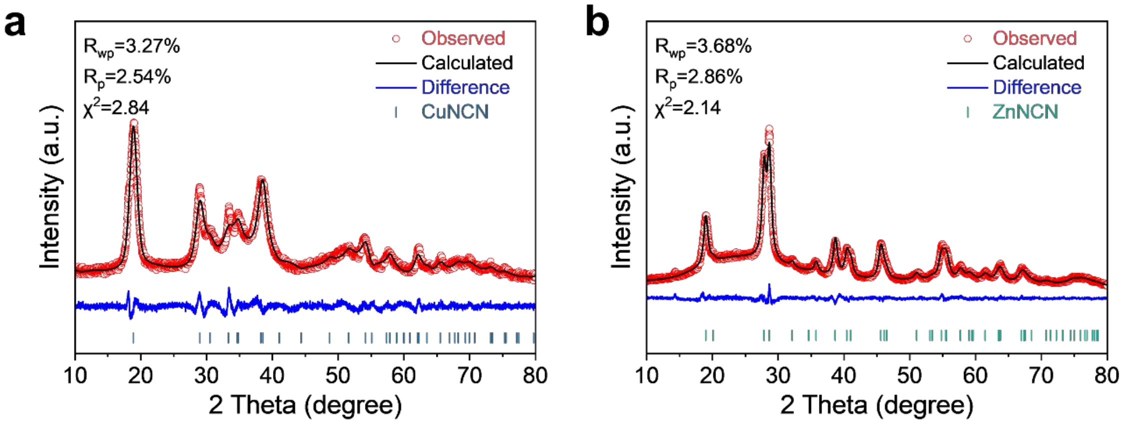

Figure S1. Refined powder X-ray diffraction data of CuNCN (a) and ZnNCN (b). The refined results unequivocally demonstrate that CuNCN adopts an Orthorhombic crystal system, whereas ZnNCN exhibits a Tetragonal crystal structure.

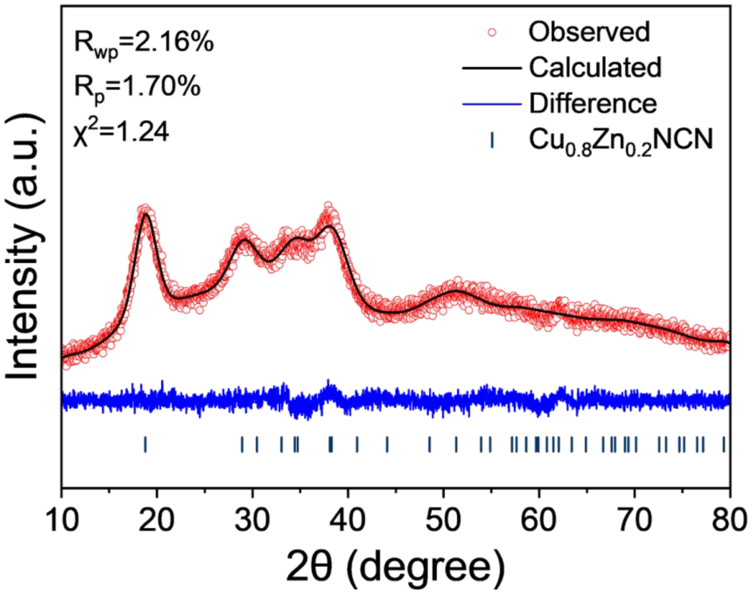

Figure S2. Refined powder X-ray diffraction data of $Cu_{0.8}Zn_{0.2}NCN$. $Cu_{0.8}Zn_{0.2}NCN$ has only one set of crystal structure at the Bragg site, and Zn replaces part of the Cu sites in it, with the Cu occupancy of 0.8 and Zn occupancy of 0.2 in the same position.

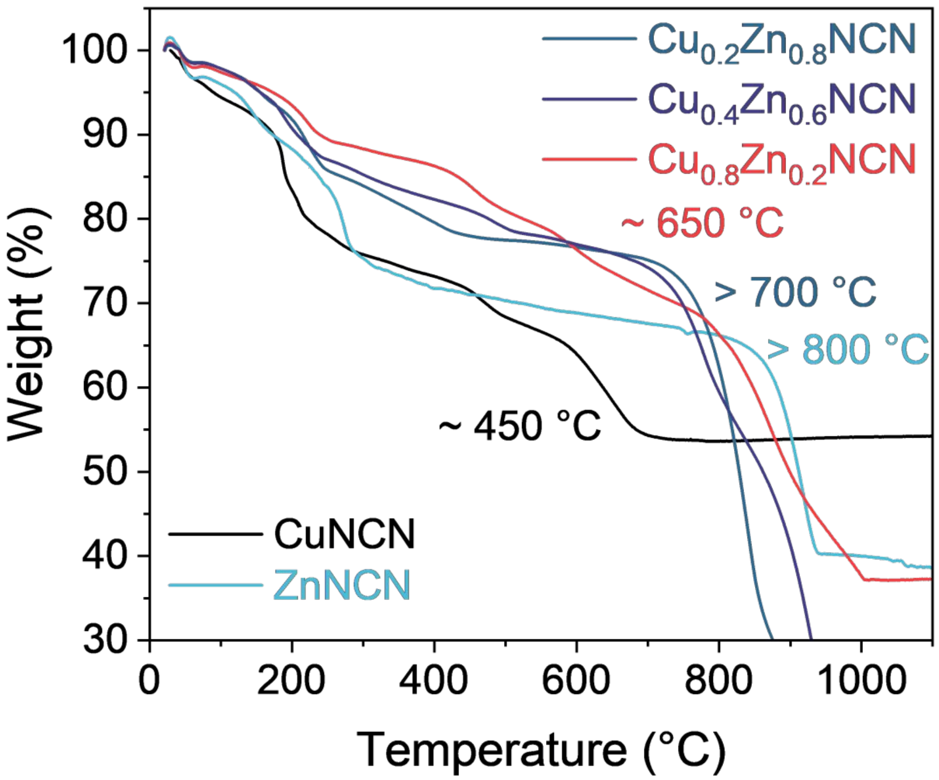

Figure S3. Thermogravimetry analysis of $Cu_{0.8}Zn_{0.2}NCN$, $Cu_{0.4}Zn_{0.6}NCN$, $Cu_{0.2}Zn_{0.8}NCN$, CuNCN and ZnNCN.

Note: The melting point of CuNCN is about 450 °C. And after the temperature exceeds 600 °C, the mass of the remaining material does not change, but the remaining substances are no longer CuNCN (may be copper carbides or nitrides instead).

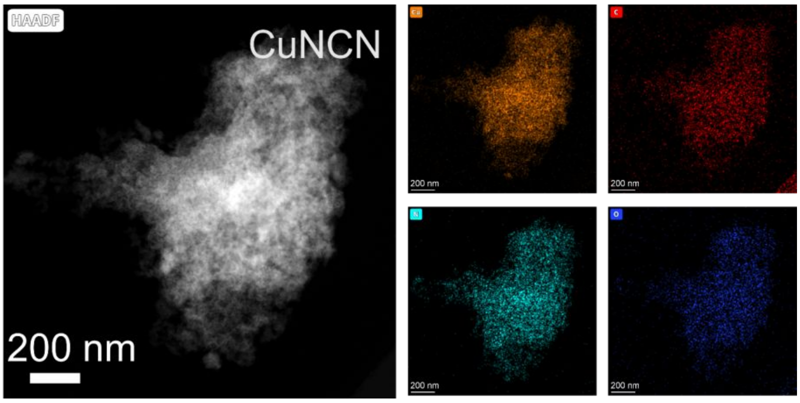

Figure S4. Structure characterization of CuNCN. TEM image and EDS mapping images of CuNCN. The EDS mapping images show the same distribution of Cu, N, and C elements in CuNCN.

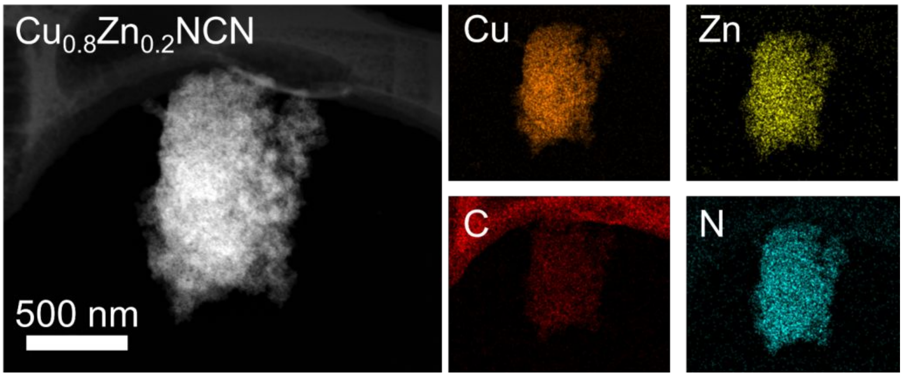

Z	Element	Family	Atomic Fraction (%)	Atomic Error (%)	Mass Fraction (%)	Mass Error (%)	Fit Error (%)
6	C	K	31.10	2.43	11.76	1.09	0.94
7	N	K	32.06	4.59	14.14	2.73	1.21
29	Cu	K	29.89	3.58	59.80	4.01	0.12
30	Zn	K	6.95	1.02	14.31	2.08	0.20

Figure S5. Structure characterization of $Cu_{0.8}Zn_{0.2}NCN$. TEM image and EDS mapping images of $Cu_{0.8}Zn_{0.2}NCN$. The EDS mapping images show the same distribution of Cu, Zn, N, and C elements in $Cu_{0.8}Zn_{0.2}NCN$. And elemental analysis shows that the ratio of Cu/Zn is 4:1, which is consistent with our feed ratio.

Note: The red color is more because the TEM test support base is carbon mesh.

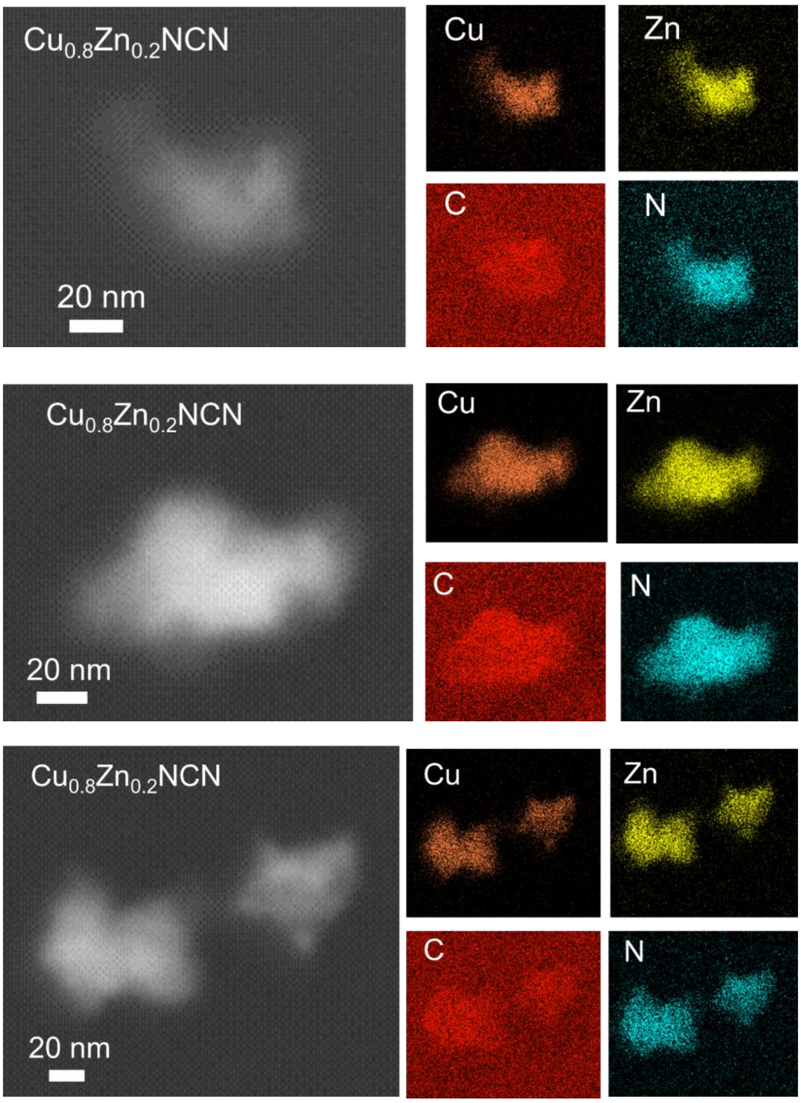

Figure S6. Structure characterization of $Cu_{0.8}Zn_{0.2}NCN$. TEM image and EDS mapping images of $Cu_{0.8}Zn_{0.2}NCN$. The EDS mapping images show the same distribution of Cu, Zn, N, and C elements in $Cu_{0.8}Zn_{0.2}NCN$. Under the condition of smaller particles, Cu and Zn can still be evenly distributed, according with the solid-solution state of $Cu_{0.8}Zn_{0.2}NCN$.

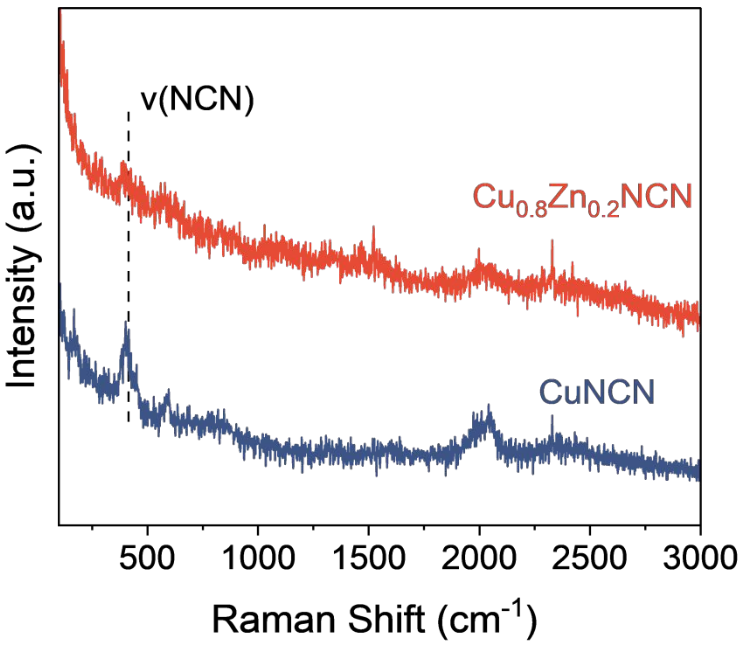

Figure S7. Raman spectra of $Cu_{0.8}Zn_{0.2}NCN$ and CuNCN.

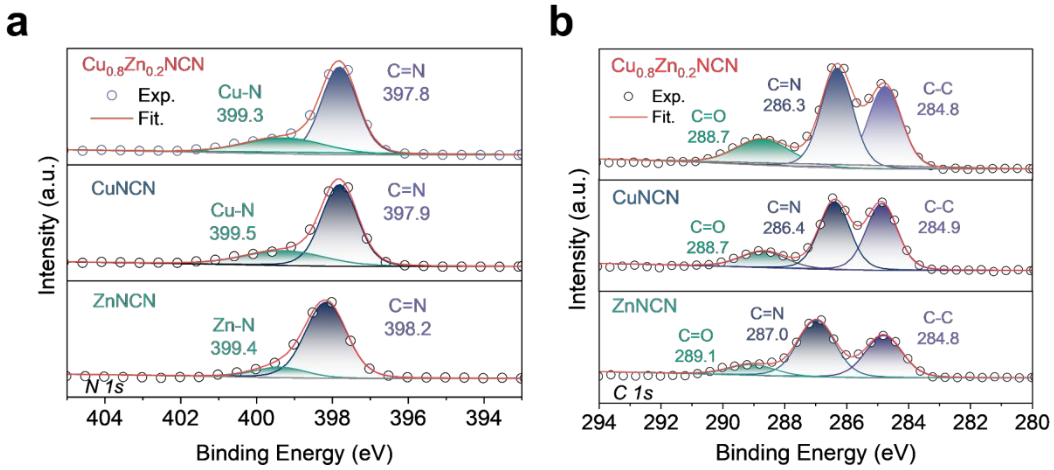

Figure S8. High-resolution X-ray photoelectron spectroscopy (XPS) of $Cu_{0.8}Zn_{0.2}NCN$, CuNCN and ZnNCN. Deconvoluted spectra of N 1s (**a**) and C 1s (**b**).

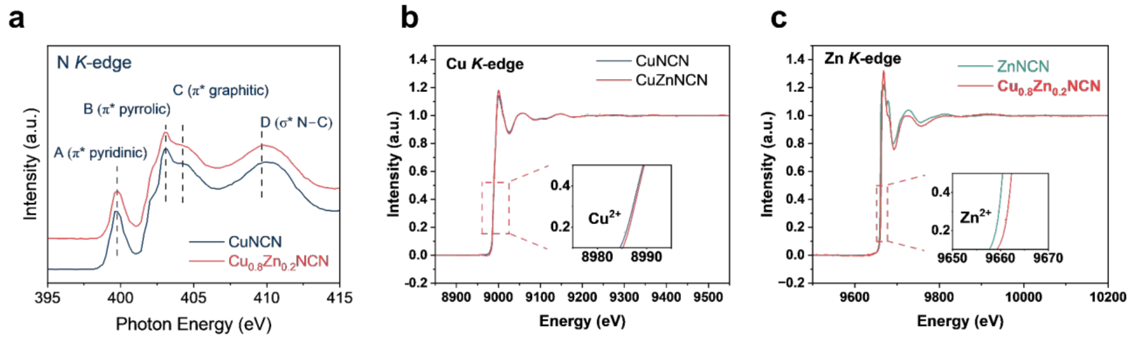

Figure S9. a, N K-edgre XAS spectra of $Cu_{0.8}Zn_{0.2}NCN$ and CuNCN. **b-c**, Line-scan spectra of $Cu_{0.8}Zn_{0.2}NCN$, CuNCN and ZnNCN. Spectra of b) Cu K edge and c) Zn K edge.

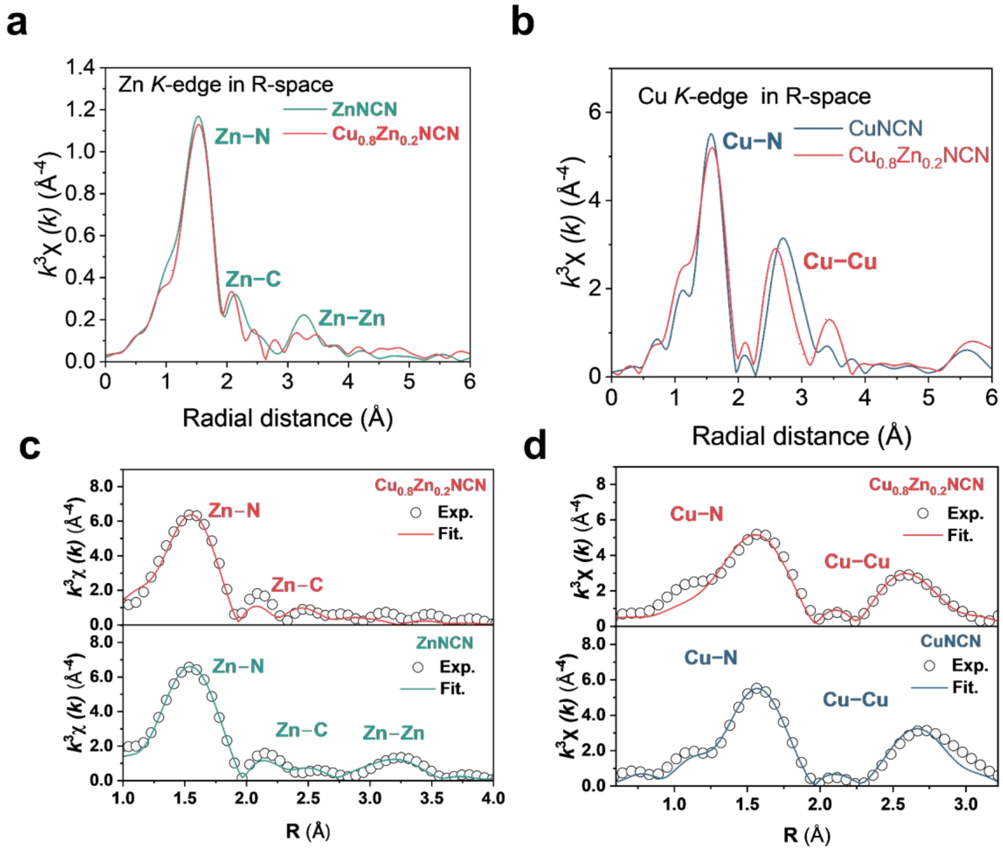

Figure S10. (a, c) Corresponding Zn K-edge EXAFS fittings of $Cu_{0.8}Zn_{0.2}NCN$ and ZnNCN. (b, d), Corresponding Cu K-edge EXAFS fittings of $Cu_{0.8}Zn_{0.2}NCN$ and CuNCN.

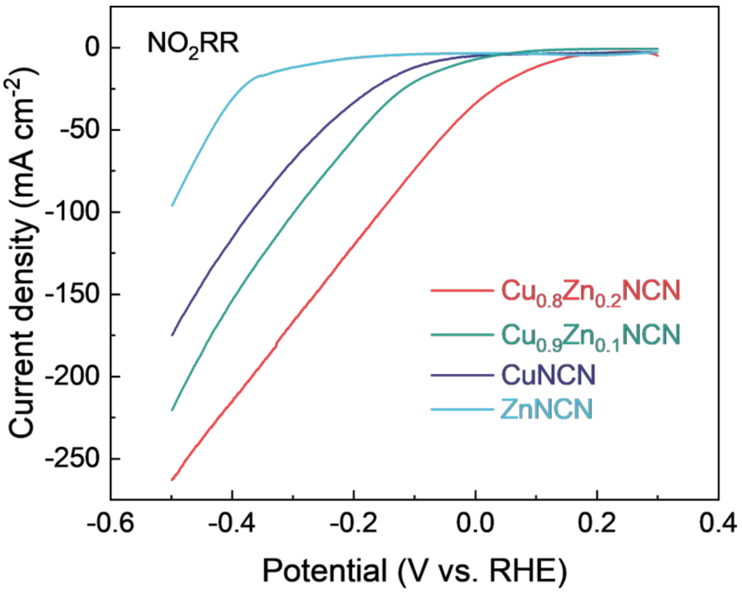

Figure S11. LSV curves of $Cu_{1-x}Zn_xNCN$. LSV curves of $Cu_{0.8}Zn_{0.2}NCN$, $Cu_{0.9}Zn_{0.1}NCN$, CuNCN, and ZnNCN.

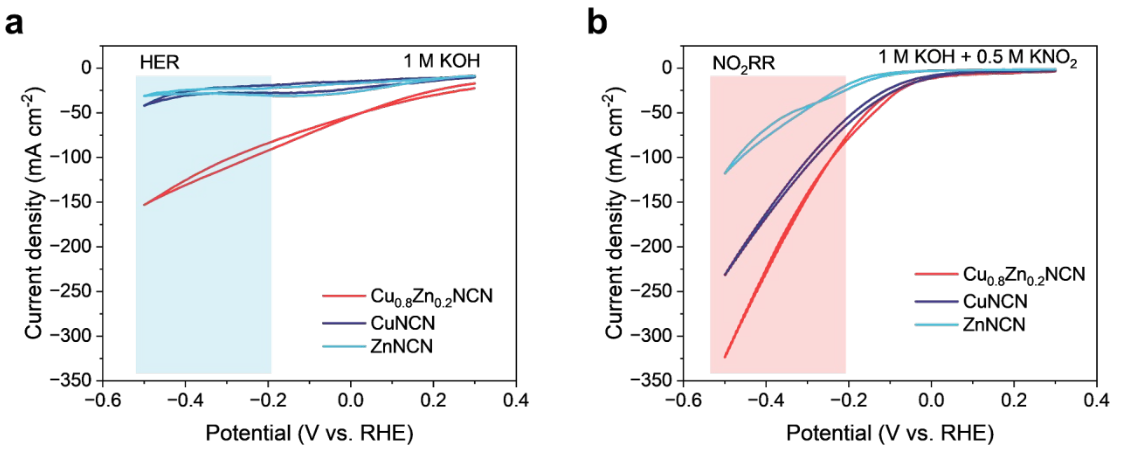

Figure S12. CV measurements. CV curves of $Cu_{0.8}Zn_{0.2}NCN$, CuNCN, and ZnNCN in 1 M KOH with or without 0.5 M KNO_2 solution.

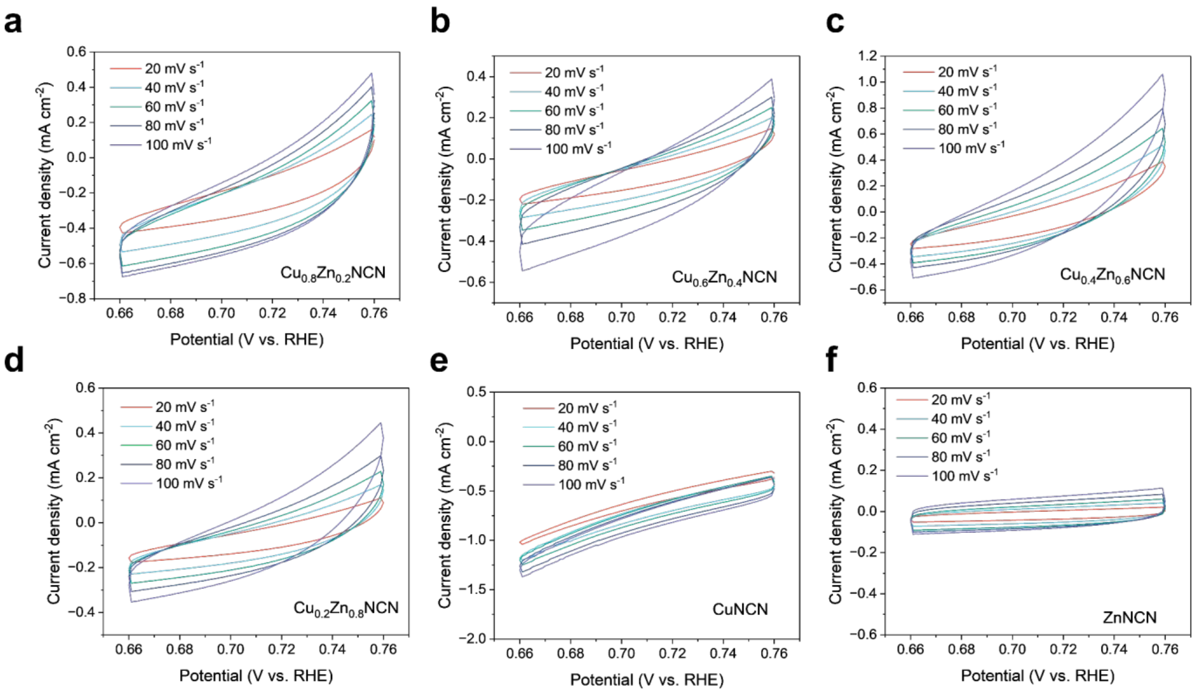

Figure S13. ECSA measurements. CV curves of $Cu_{0.8}Zn_{0.2}NCN$ (a), $Cu_{0.6}Zn_{0.4}NCN$ (b), $Cu_{0.4}Zn_{0.6}NCN$ (c), $Cu_{0.2}Zn_{0.8}NCN$ (d), CuNCN (e), and ZnNCN (f) with different scan rates of 20, 40, 60, 80, and 100 mV s^{-1}.

252

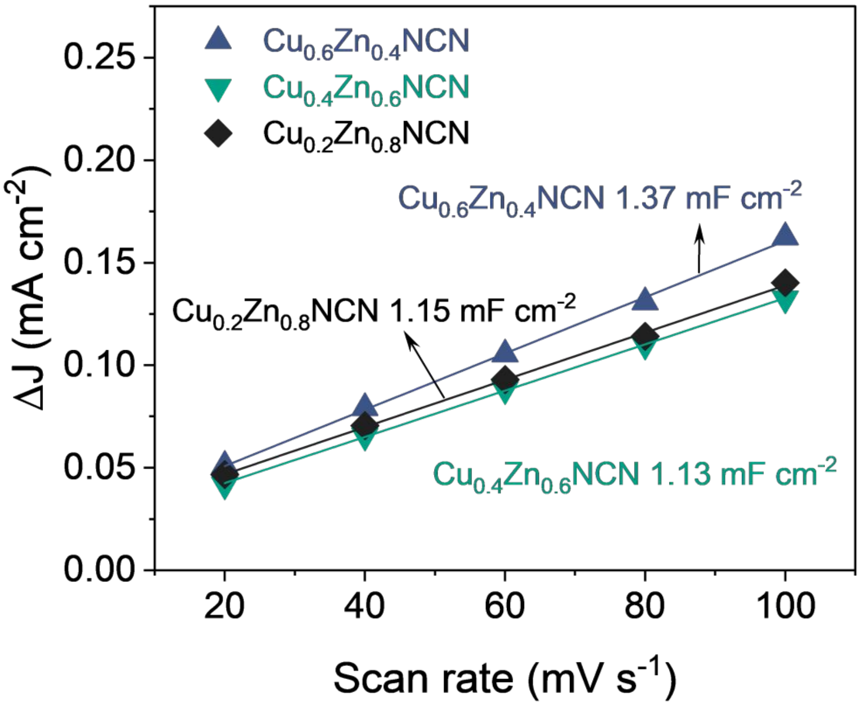

253

254 **Figure S14.** ECSA measurements of $Cu_{0.6}Zn_{0.4}NCN$, $Cu_{0.4}Zn_{0.6}NCN$, and $Cu_{0.2}Zn_{0.8}NCN$.

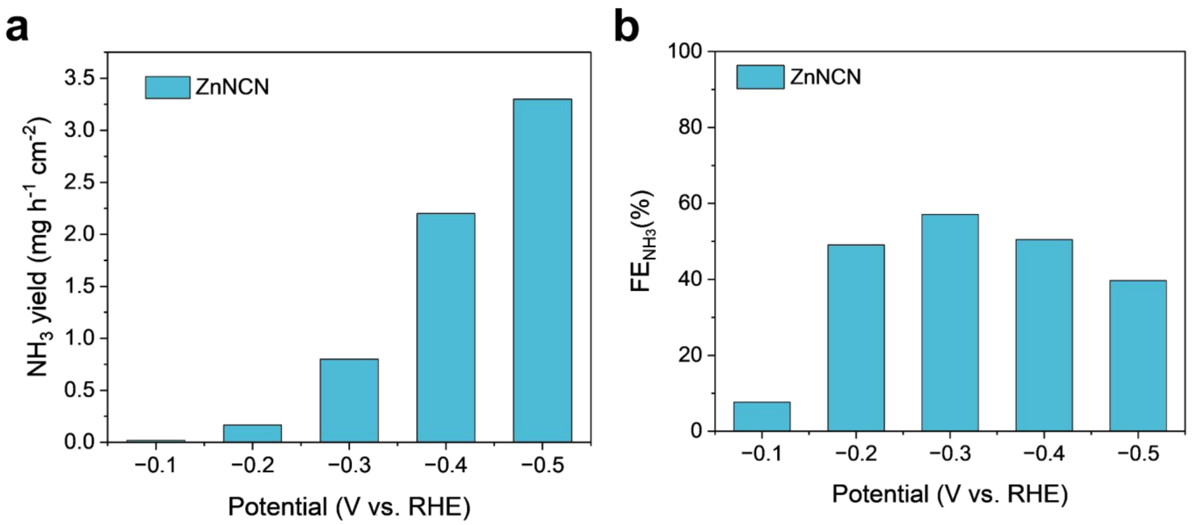

Figure S15. NH_3 yield and Faradaic efficiency for ZnNCN at the given potentials of NO_2RR.

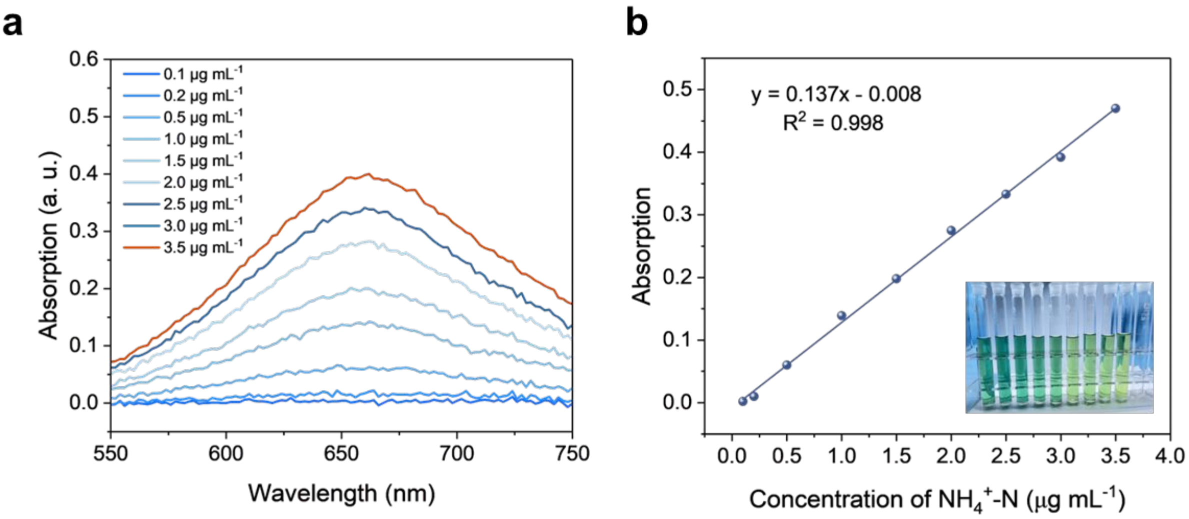

Figure S16. (a) UV-vis calibration curve of NH_3 in ultrapure water using NH_4Cl solutions of known concentration as standards. **(b)** Calibration curve used for estimation of NH_3.

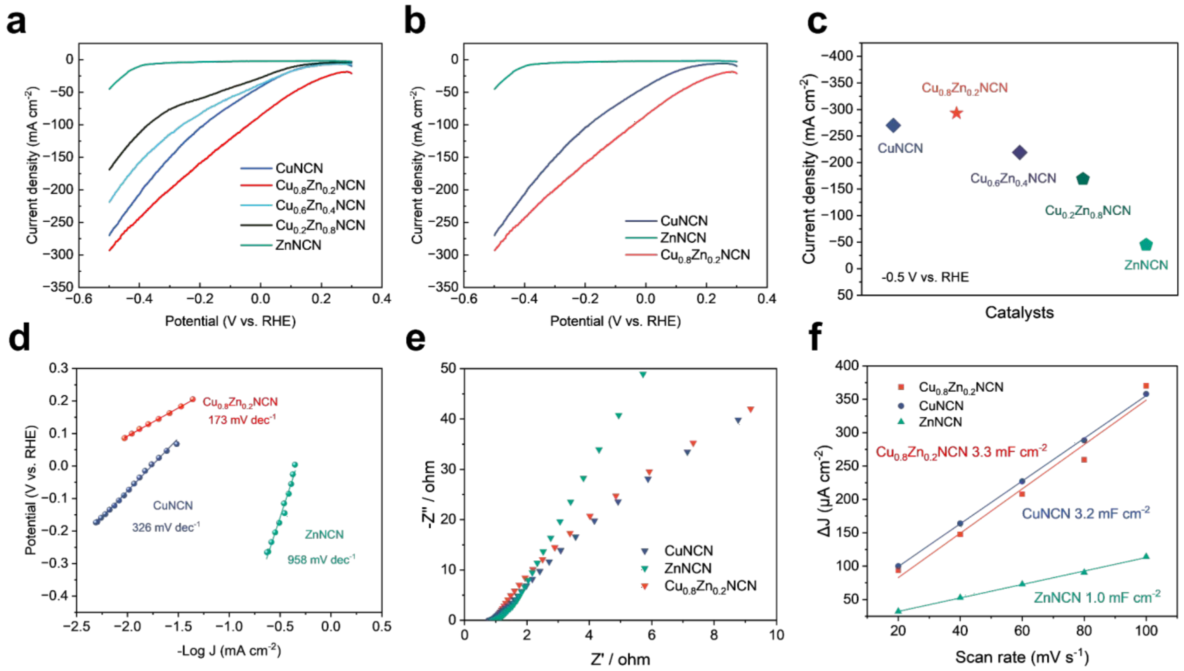

Figure S17. Electrochemical measurements of NO_3RR for various samples.

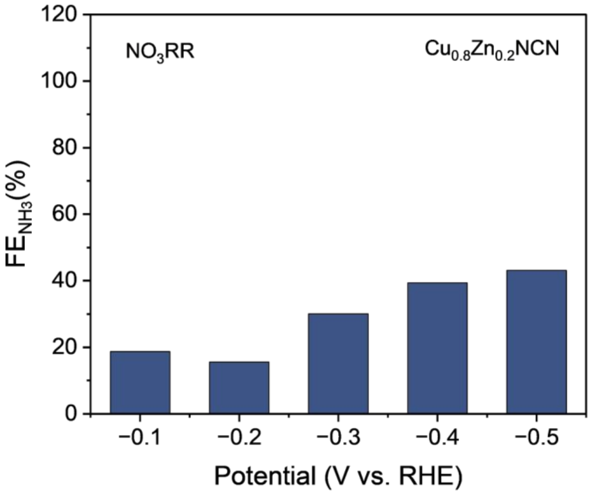

Figure S18. NH_3 Faradaic efficiency for $Cu_{0.8}Zn_{0.2}NCN$ at the given potentials of NO_3RR.

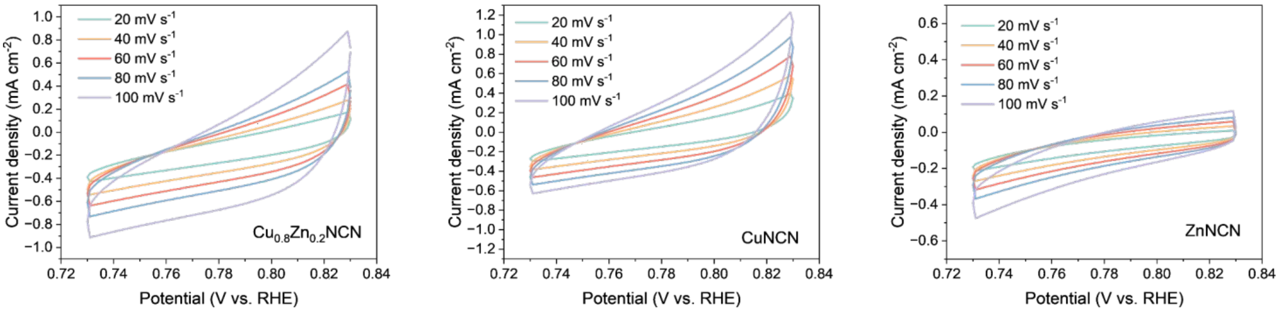

Figure S19. ECSA measurements of NO_3RR for $Cu_{0.8}Zn_{0.2}$NCN, CuNCN, and ZnNCN

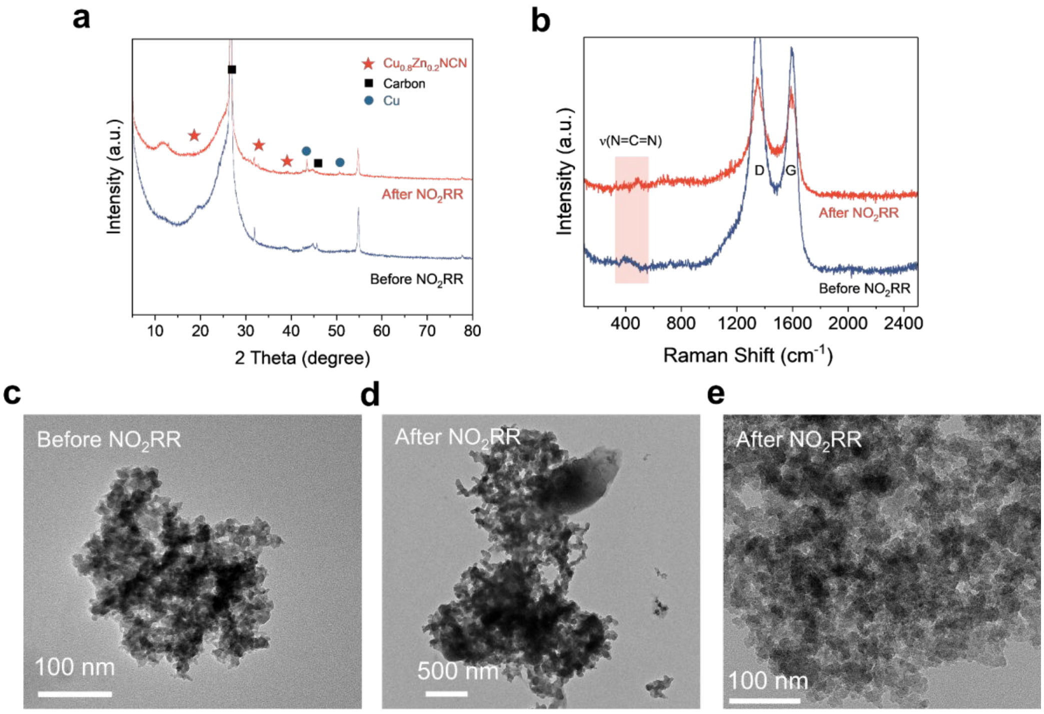

Figure S20. Structure and morphology of $Cu_{0.8}Zn_{0.2}NCN$ before and after the NO_2RR electrolysis. (a) XRD pattern, (b) Raman spectra, and (c-e) TEM images of $Cu_{0.8}Zn_{0.2}NCN$.

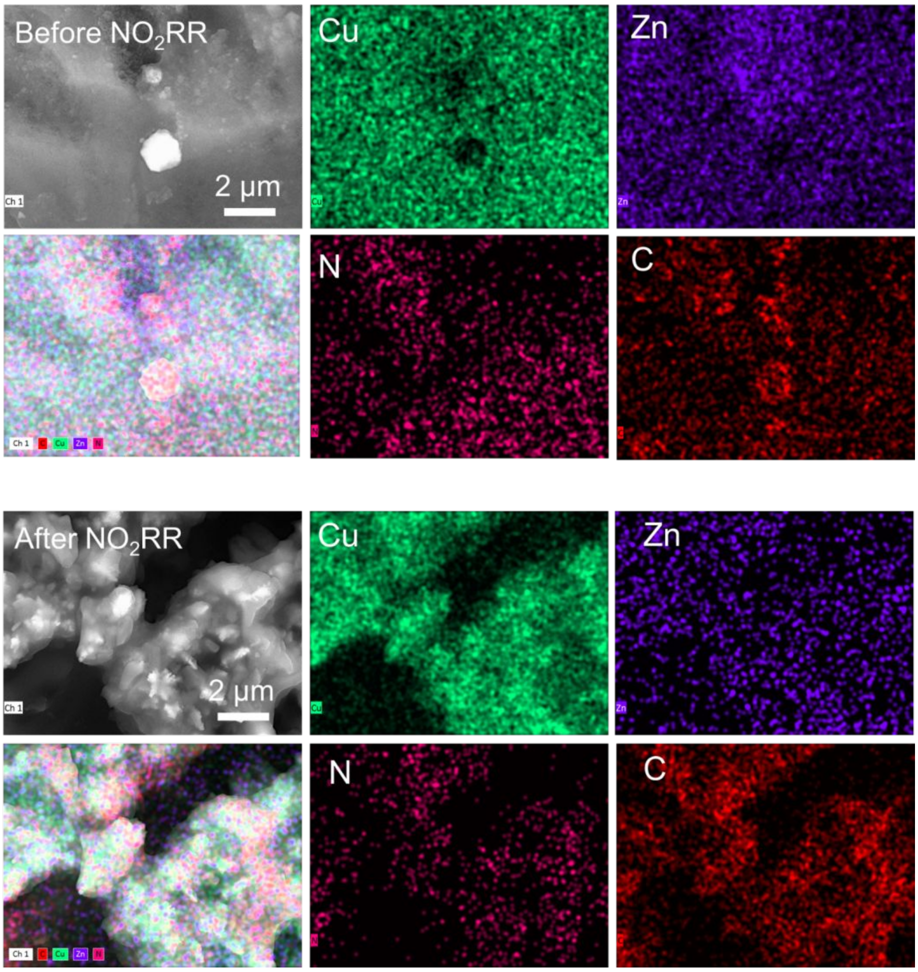

Figure S21. SEM images $Cu_{0.8}Zn_{0.2}NCN$ before and after the NO_2RR electrolysis.

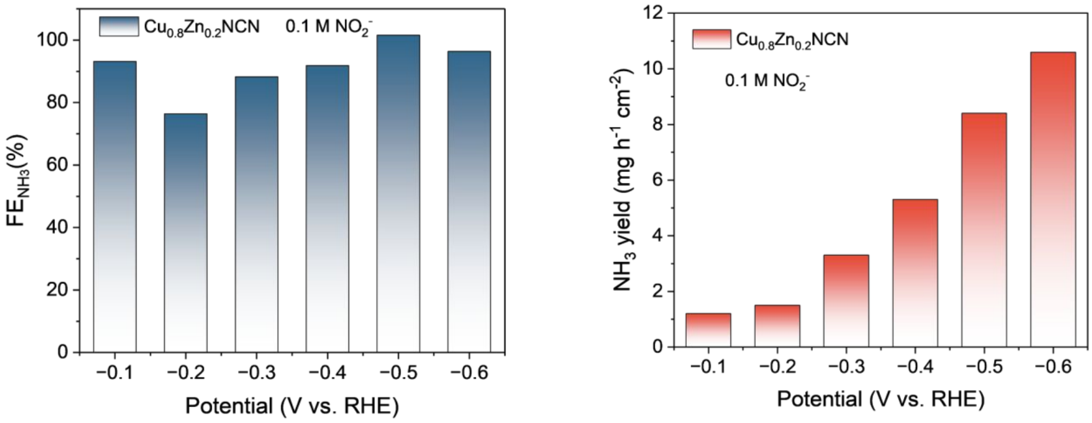

Figure S22. NH_3 Faradaic efficiency (a) and NH_3 yields (b) for $Cu_{0.8}Zn_{0.2}NCN$ at the given potentials 0.1 M NO_2^- electrolyte.

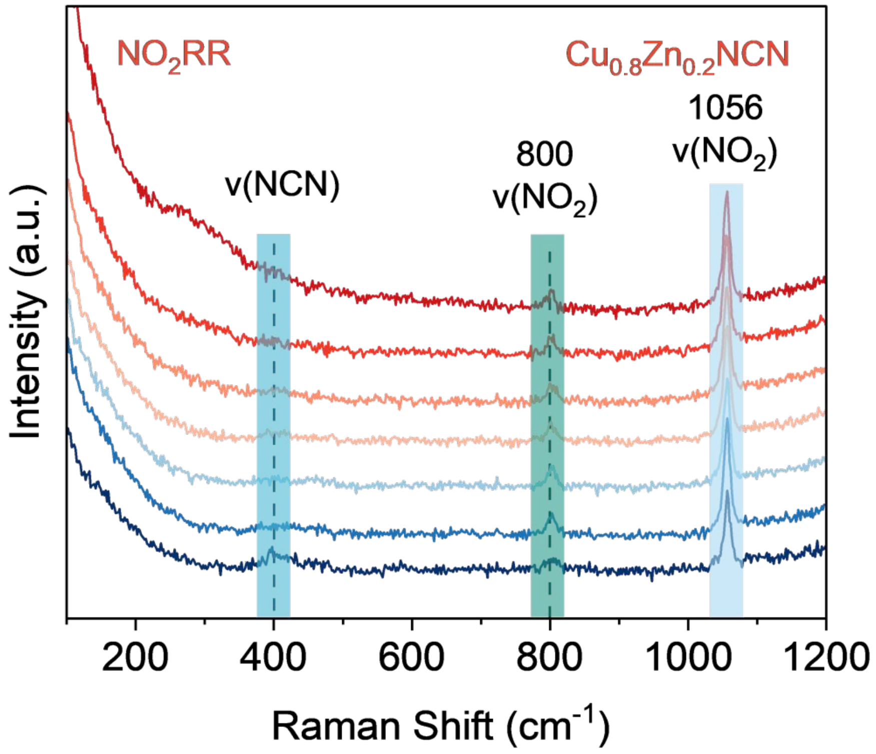

Figure 23. In-situ Raman spectra (100−1200 cm^{-1}) of $Cu_{0.8}Zn_{0.2}NCN$ in NO_2RR at a potential range from OCP to −0.2 V (vs. RHE).

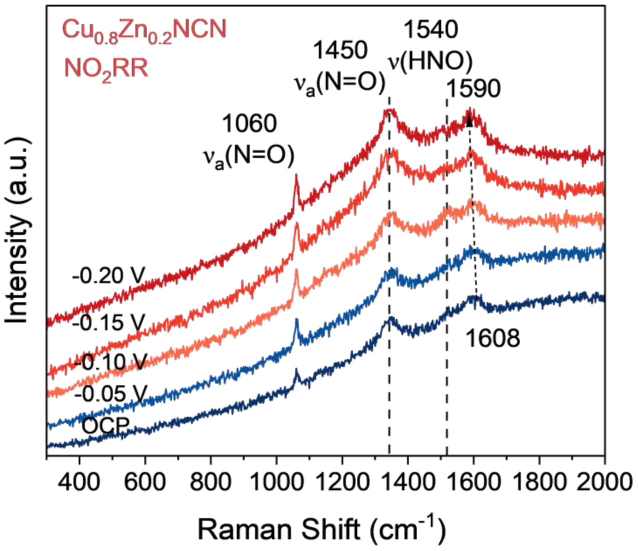

Figure 24. In-situ Raman spectra (300−2000 cm^{-1}) of $Cu_{0.8}Zn_{0.2}NCN$ in NO_2RR at a potential range from OCP to −0.20 V (vs. RHE).

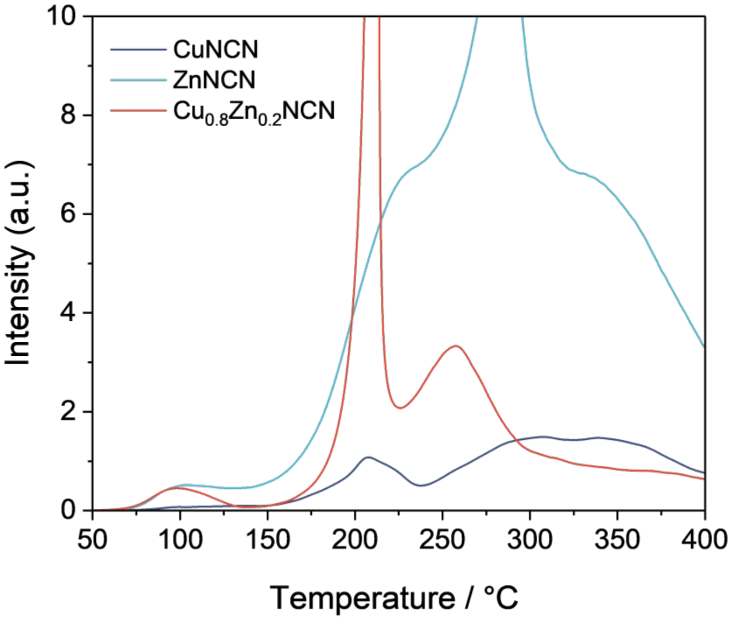

Figure S25. NO-TPD curves of pristine $Cu_{0.8}Zn_{0.2}NCN$, CuNCN and ZnNCN nanocrystals. $Cu_{0.8}Zn_{0.2}NCN$ exhibits greater adsorption of NO than CuNCN and ZnNCN.

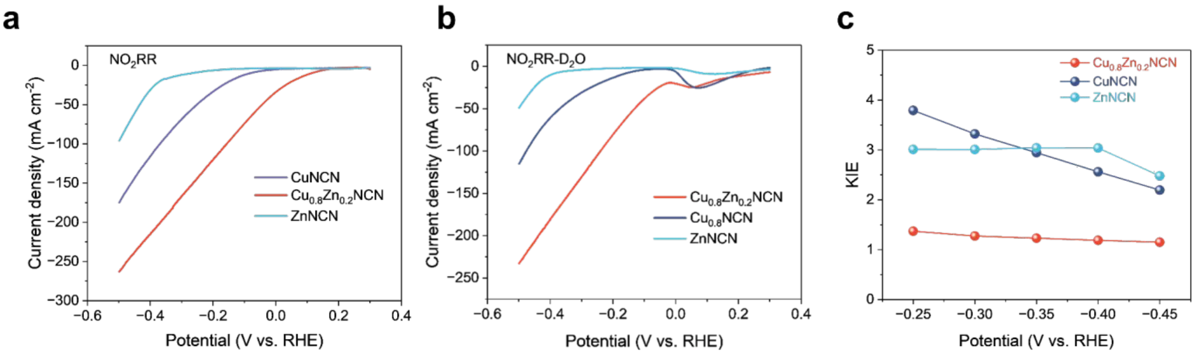

Figure S26. The LSV curves from $Cu_{0.8}Zn_{0.2}NCN$, CuNCN, and ZnNCN catalysts in the H_2O-based and D_2O-based electrolyte containing 1.0 M KOH with and without 0.5 M KNO_3.

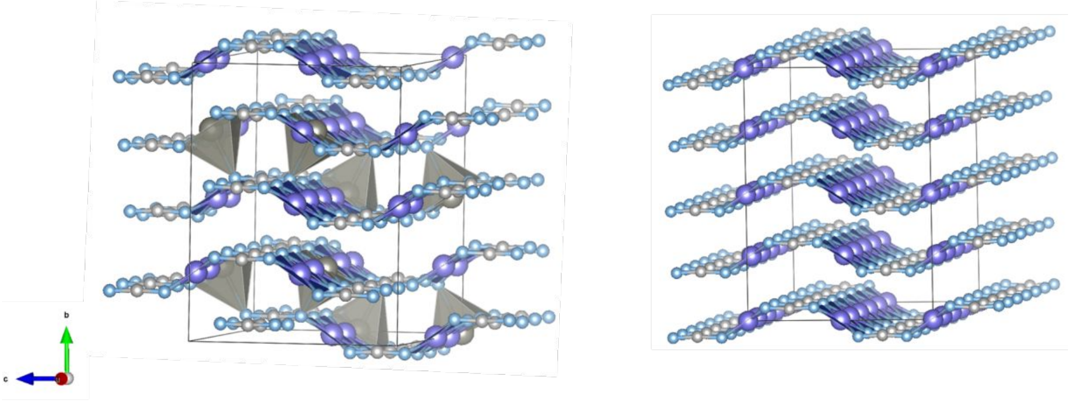

$Cu_{0.8}Zn_{0.2}NCN$ CuNCN

Figure S27. Schematic crystal structure of $Cu_{0.8}Zn_{0.2}NCN$ and CuNCN.

Considering the bimetallic effect on the electronic structure of cyanamide compound materials, we primarily investigated the electronic structure and performance of CuNCN and $Cu_{0.8}Zn_{0.2}NCN$ models through DFT calculations and electrochemical tests.

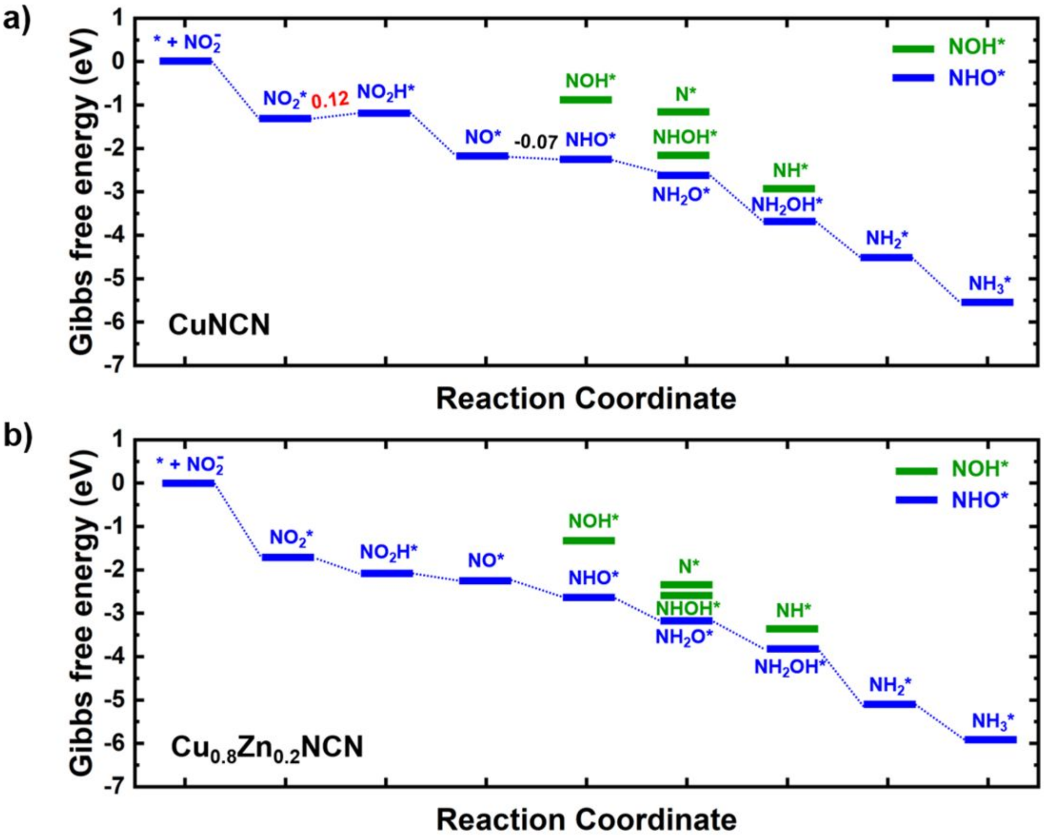

Figure S28. Free-energy diagrams for all possible reaction pathways of NO_2^- reduction on a) CuNCN and b) $Cu_{0.8}Zn_{0.2}NCN$. The blue and green bars demonstrate the NHO* and NOH* paths, respectively. Color code: Cu – blue, Zn – silver, C – brown, N – light blue, O – red and H – light pink. Unit: eV

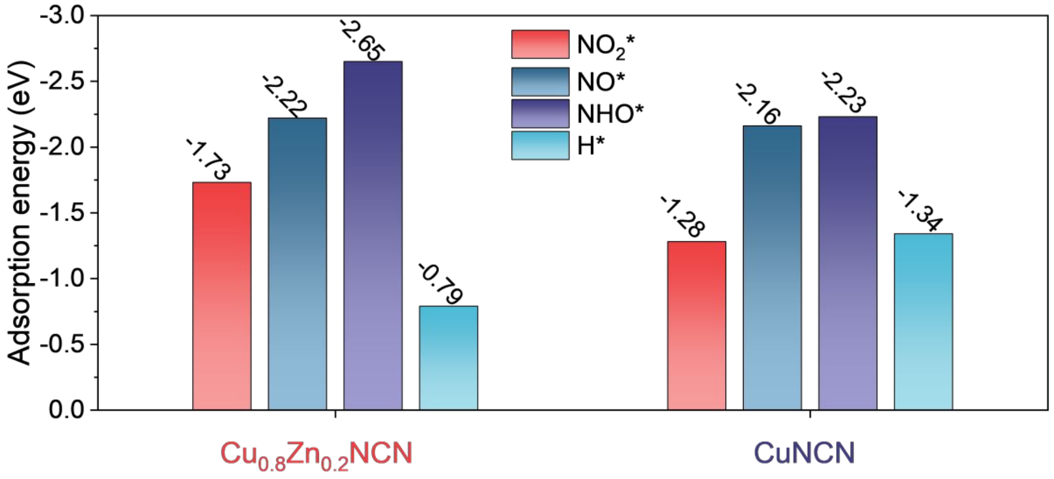

Figure S29. The calculated adsorption energies of different species on $Cu_{0.8}Zn_{0.2}NCN$ and CuNCN.

By calculating the adsorption energies of $Cu_{0.8}Zn_{0.2}NCN$ and CuNCN electrocatalysts, we observe that $Cu_{0.8}Zn_{0.2}NCN$ exhibits the greater adsorption energy of NO_2*, NO*, NHO*, and moderate adsorption of H*.

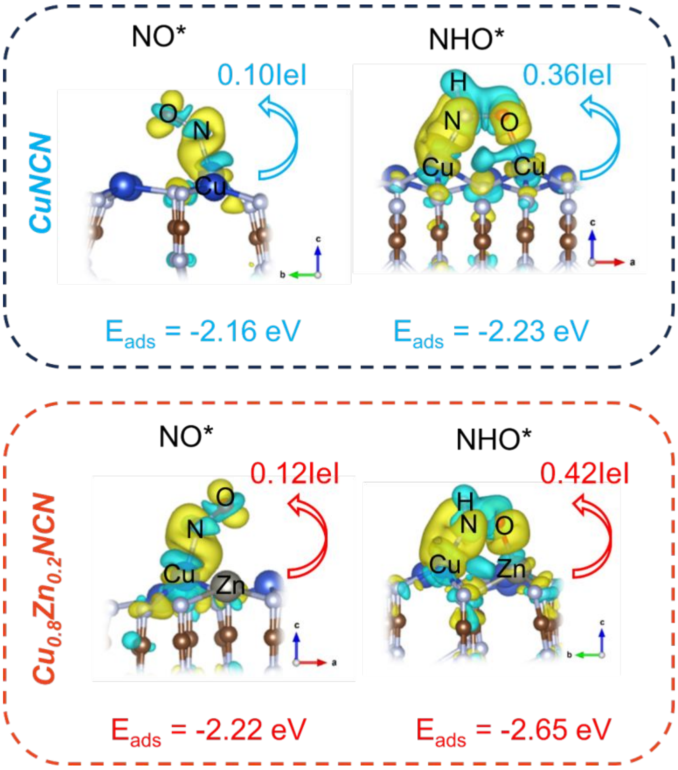

Figure S30. Charge density difference and adsorption energy for NO* and NHO* adsorption on CuNCN (upper) and $Cu_{0.8}Zn_{0.2}NCN$ (down).

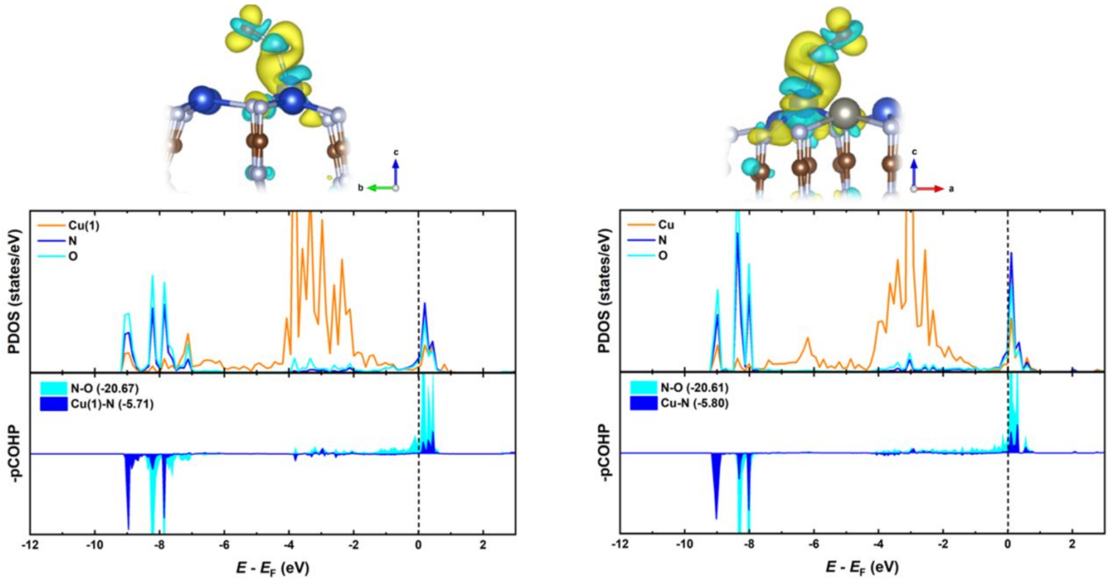

Figure S31. Partial density of states (PDOS) of surface atoms and adsorbed NO* species (upper panels); and projected crystal orbital Hamilton population (pCOHP) (lower panels) within NO* intermediate. Their corresponding charge density difference are also displayed. The isosurface value is set to 0.002 e/Å^3, and the yellow (cyan) regions represent charge accumulation (depletion). The Fermi level displayed in dashed line is set to zero.

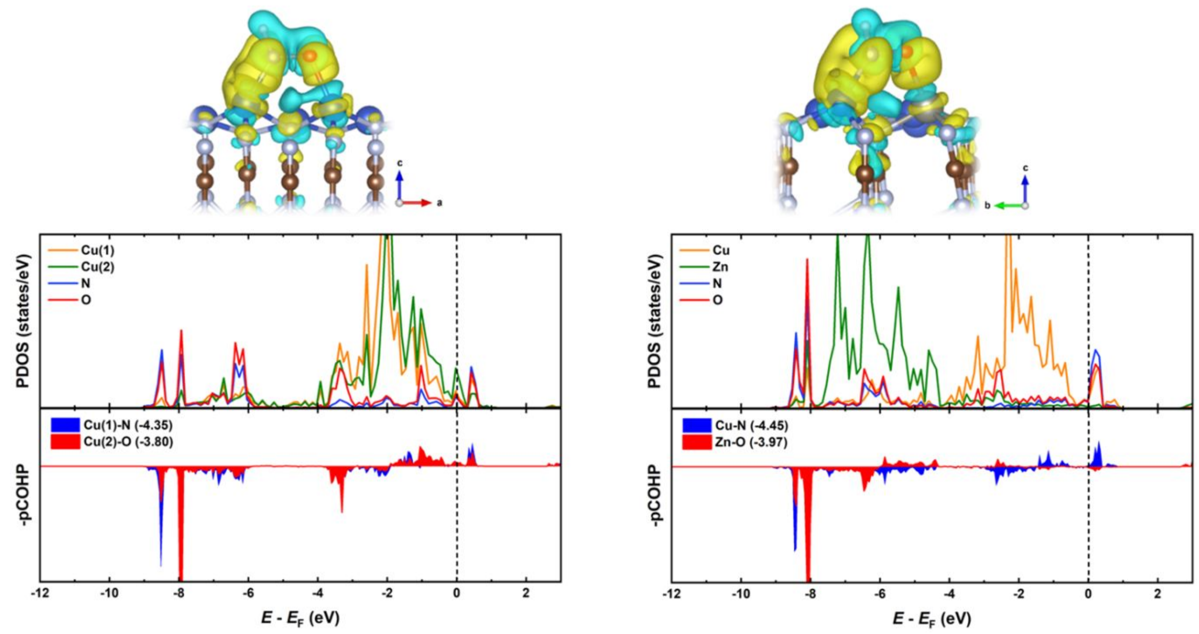

Figure S32. Partial density of states (PDOS) of surface atoms and adsorbed NHO* species (upper panels); and projected crystal orbital Hamilton population (pCOHP) (lower panels) within NHO* intermediate. Their corresponding charge density difference are also displayed. The isosurface value is set to 0.002 e/Å^3, and the yellow (cyan) regions represent charge accumulation (depletion). The Fermi level displayed in dashed line is set to zero.

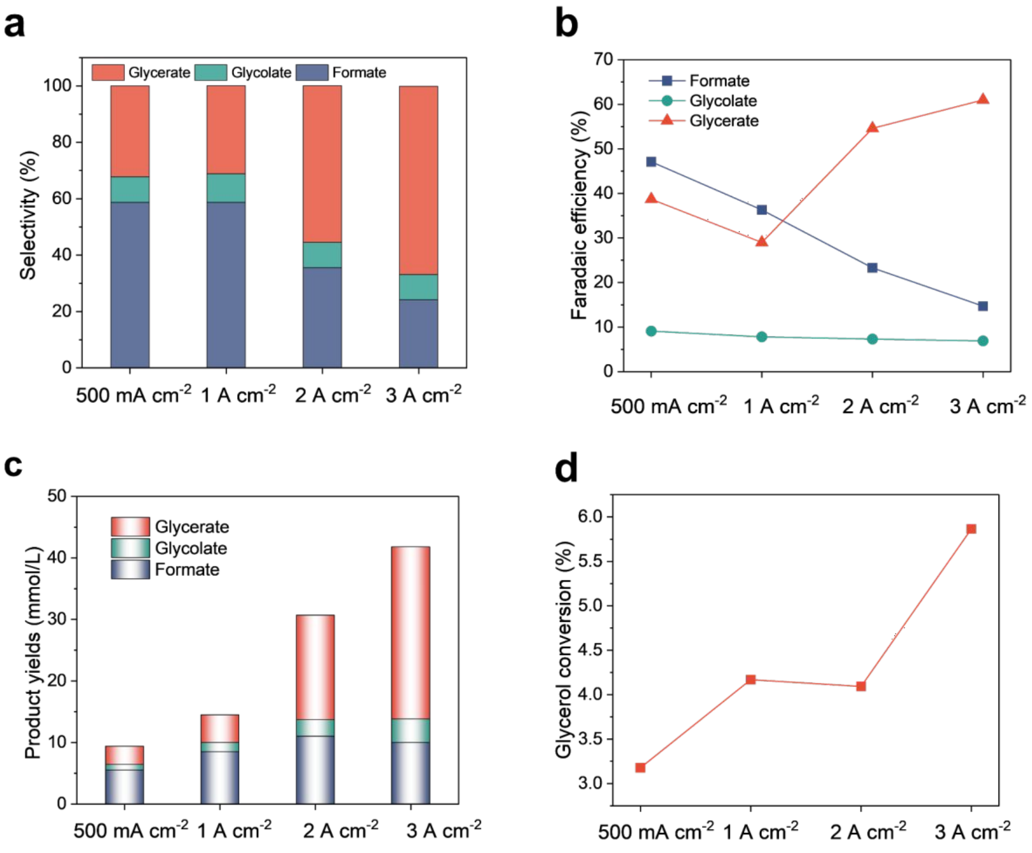

Figure S33. The product selectivity, product Faradaic efficiency, and glycerol conversion of anodic glycerol electrooxidation in the membrane electrode assembly. The product selectivity (**a**), product Faradaic efficiency (**b**), product yields (**c**), and glycerol conversion (**d**) of anodic glycerol electrooxidation at different current density (0.5, 1, 2, 3 A cm^{-2}) for 10 min.

Supplementary Tables

Table S1. Crystallographic information for the studied CuNCN and ZnNCN. Reported lattice parameters are given in square brackets.

Phase	CuNCN	ZnNCN
Crystal system	Orthorhombic	Tetragonal
Space group	Cmcm	I-42d
a[Å]	2.9874(6)	8.7740(6)
b[Å]	6.1602(13)	8.7740
c[Å]	9.4034(14)	5.4660(4)
V[Å^3]	173.05(5)	420.79(5)

The refined results unequivocally demonstrate that CuNCN adopts an Orthorhombic crystal system, whereas ZnNCN exhibits a Tetragonal crystal structure.

Table S2. Crystallographic information for the studied $Cu_{0.8}Zn_{0.2}NCN$. Reported lattice parameters are given in square brackets.

**Phase**	**CuNCN**	**$Cu_{0.8}Zn_{0.2}NCN$**
Crystal system	Orthorhombic	Orthorhombic
Space group	Cmcm	Cmcm
a[Å]	2.99205	3.01418
b[Å]	6.17821	6.16891
c[Å]	9.40031	9.43613
V[$Å^3$]	173.77	175.46

The solid solution $Cu_{0.8}Zn_{0.2}NCN$ has only one set of crystal structure at Bragg site, and zinc replaces part of the copper in it, with the copper occupancy of 0.8 and zinc occupancy of 0.2 in the same position (**Figure S2**). The $Cu_{0.8}Zn_{0.2}NCN$ still maintains the orthorhombic system and Cmcm space group, which is consistent with CuNCN. Since the atomic radius of zinc (1.38 Å) is larger than that of copper (1.28 Å), the lattice expands slightly after the introduction, which further proves the successful introduction of zinc.

Table S3. ICP-OES analysis of $Cu_{0.8}Zn_{0.2}NCN$

Sample	Cu (mg/L)	Zn (mg/L)	Cu/Zn ratio
**$Cu_{0.8}Zn_{0.2}NCN$**	1309.0	312.2	4.2
	1181.0	310.5	3.8

The results show that the atomic ratios of Cu : Zn in the products are consistent with the intended feed ratios, confirming that the metal distribution is preserved in the final copper-zinc cyanamide material. The measured ratio of Cu : Zn in the $Cu_{0.8}Zn_{0.2}NCN$ sample is 4.2 : 1 or 3.8 : 1, which closely matches the targeted ratio.

Table S4. EXAFS analyses.

Fitting results (i.e., as-acquired values of structural parameters) for the first coordination shell (1–3 Å) of Cu Atoms in CuNCN, ZnNCN, and $Cu_{0.8}Zn_{0.2}NCN$ samples from Cu K-edge EXAFS Data. (ΔE0: energy shift; CN: coordination number; ⟨R⟩: interatomic distance; σ^2: Debye-Waller factor).

Sample	Fit path	CN	ΔE (eV)	R (Å)	σ^2 ($Å^2$)	R factor
**CuNCN**	Cu−N	4.0 (set)	5.8±1.3	1.984±0.011	0.005±0.002	0.026
	Cu−Cu	1.6±0.7	5.8±1.3	2.966±0.013	0.005±0.002	0.026
**$Cu_{0.8}Zn_{0.2}NCN$**	Cu−N	4.8±0.4	4.5±1.7	1.981±0.013	0.006±0.002	0.019
	Cu−Cu	1.6±0.8	4.5±1.7	2.941±0.017	0.006±0.004	0.019
**ZnNCN**	Zn−N	4.0 (set)	4.3±0.6	1.977±0.005	0.005±0.002	0.022
	Zn−C	2.1±0.7	4.3±0.6	3.204±0.037	0.008 (set)	0.022
	Zn−Zn	1.8±0.3	4.3±0.6	3.394±0.026	0.010 (set)	0.022
**$Cu_{0.8}Zn_{0.2}NCN$**	Zn−N	4.0±0.2	2.1±0.5	1.985±0.010	0.007±0.002	0.017
	Zn−C	1.2±1.0	2.1±0.5	3.174±0.036	0.008±0.012	0.017

Note: EXAFS is not able to distinguish bond length less than 0.1 A difference. The bond length of one sample represents an average value. The amplitude reduction factor was set to be 0.75.

Table S5. NO_2RR **performance comparison for various electrocatalysts.**

Catalysts	Electrolyte	FE_{NH3}(%)	Potential (V vs. RHE)	NH_3 yield rate ($mg\ h^{-1}\ cm^{-2}$)	Current ($mA\ cm^{-2}$)	Ref.
**$Cu_{0.8}Zn_{0.2}N$ CN**	**0.5 M NO_2^-, 1 M KOH**	**~100**	**-0.5**	**22.0 mg h^{-1} cm^{-2}**	**280**	**This work**
**$Cu_{0.8}Zn_{0.2}N$ CN**	**0.1 M NO_2^-, 1 M KOH**	**~100**	**-0.5**	**8.4 mg h^{-1} cm^{-2}**	**96**	**This work**
$Co_{0.5}NiS$-NSs/NF	0.05 M NO_2^-, 1 M KOH	92.2	**-0.15**	4.25 mg h^{-1} cm^{-2}	105	10
Ag@NiO/CC	0.1 M NO_2^-, 0.1 M KOH	97.7	**-0.5**	5.75 mg h^{-1} cm^{-2}	53	11
Fe-N-C single-atom	0.01 M NO_2^- 0.05 M PBS	100	**-0.7**	0.86 mg h^{-1} cm^{-2}	60	12
CoP nanoarrays	500 ppm NO_2^- 0.1 M PBS	90.0	**-0.2**	2.30 mg h^{-1} cm^{-2}	~60	13
Cu_3P NA/CF	0.1 M NO_2^- 0.1 M PBS	95.7	**-0.5**	4.08 mg h^{-1} cm^{-2}	~120	14
Ni_2P/NF	200 ppm NO_2^- 0.1 M PBS	90.2	**-0.3**	3.25 mg h^{-1} cm^{-2}	~98	15
Ni@MDC	0.1 M NO_2^-, 0.1 M NaOH	65.4	**-0.8**	5.10 mg h^{-1} cm^{-2}	~90	16
V-doped	0.1 M NO_2^-	93.2	**-0.7**	9.18 mg h^{-1}	95	17

TiO_2	0.1 M NaOH			cm^{-2}		
Pd/CuO nano-olives	0.01 M NO_2^-, 0.1 M K_2SO_4	94.9	**-1.5**	0.91 mg h^{-1} mg^{-1}	65	18
Cu nanoparticles	0.1 M NO_2^-, 0.1 M KOH	93.2	**-0.6**	8.9 mg h^{-1} mg^{-1}	90	19
Ni-JBC	0.1 M NO_2^-, 0.1 M KOH	83.4	**-0.5**	4.12 mg h^{-1} mg^{-1}	85	20
WO_2 nanoparticles	0.1 M NO_2^- 0.1 M KOH	94.3	**-0.9**	15.0 mg h^{-1} cm^{-2}	140	21
Co nanoparticles	0.1 M NO_2^-, 0.1 M KOH	96.9	**-1.0**	2.8 mol h^{-1} g_{Co}^{-1}	75	22

Table S6. Bulk structures of three types structures analyses.

$Cu_{0.8}Zn_{0.2}NCN$	1	2	3	4	5	6	7
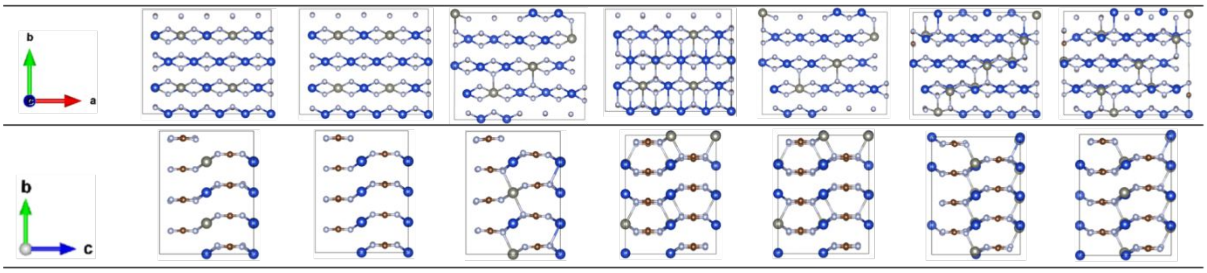							
Total Energy	-1135.991	-1137.156	-1136.431	-1136.324	-1137.607	-1136.631	-1136.587
$Cu_{0.8}Zn_{0.2}NCN$	8	9	10	11	12	CuNCN	
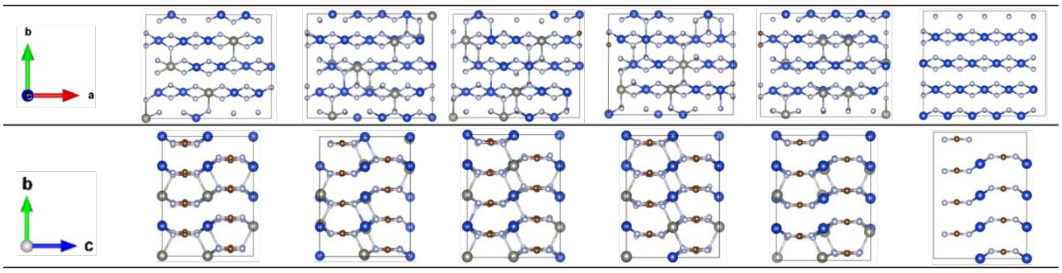							
Total Energy	-1137.644	-1136.646	-1136.732	-1136.367	-1136.485	-1149.019	

Among bulk structures of three types structures, model No.8 is chosen for $Cu_{0.8}Zn_{0.2}NCN$ based on the lowest total energy.

Table S7. Mulliken charge variations of active atoms in two surfaces (CuNCN and $Cu_{0.8}Zn_{0.2}NCN$) and adsorbed atoms within three kinds of intermediates (NO_2*, NO* and NHO*. Total Mulliken charge adsorbed species are calculated in each intermediate. Unit: |e|.

Mulliken charge		Surfaces		Adsorbates			Total
NO_2*	CuNCN	Cu(1)	Cu(2)	N	O(1)	O(2)	NO_2*
		0.74	0.72	0.33	-0.40	-0.40	-0.47
	$Cu_{0.8}Zn_{0.2}NCN$	Cu(1)	Zn	N	O		
		0.82	1.14	0.21	-0.45		-0.54
NO*	CuNCN	Cu(1)		N	O		NO*
		0.85		0.05	-0.15		-0.10
	$Cu_{0.8}Zn_{0.2}NCN$	Cu(1)		N	O		
		0.98		0.03	-0.15		-0.12
NHO*	CuNCN	Cu(1)	Cu(2)	N	O		NHO*
		0.84	0.81	-0.35	-0.38		-0.36
	$Cu_{0.8}Zn_{0.2}NCN$	Cu(1)	Zn	N	O		
		0.93	1.11	-0.37	-0.42		-0.42

Three above key species (NO_2*, NO* and NHO*) prefer to adsorb on Cu-Zn active sites ($Cu_{0.8}Zn_{0.2}NCN$): closely associated with more amount of charge acceptance; suggesting **more effective charge transfer of the $Cu_{0.8}Zn_{0.2}NCN$ catalyst** compared to its counterpart.

Table S8. The evolution of Faradaic efficiency as a function of applied current densities.

Product	Formate (mol/L)	Glycolate (mol/L)	Glycerate (mol/L)	Formate FE(%)	Glycolate FE(%)	Glycerate FE(%)
**GOR-500 mA cm^{-2}**	0.0055	0.0009	0.0030	47.1	9.1	38.7
**GOR-1 A cm^{-2}**	0.0085	0.0015	0.0045	36.3	7.8	29.0
**GOR-2 A cm^{-2}**	0.0109	0.0027	0.0170	23.3	7.3	54.6
**GOR-3 A cm^{-2}**	0.0103	0.0038	0.0284	14.7	6.9	61.0

References

(1) G. Kresse, J. F. È., Efficient Iterative Schemes for Ab Initio Total-Energy Calculations Using a Plane-Wave Basis Set. *Physical Review B* **1996,** *54*, 11169-11186.

(2) John P. Perdew, K. B., Matthias Ernzerhof, Generalized Gradient Approximation Made Simple. *Physical Review Letters* **1996,** *77* (18), 3865-3868.

(3) Blöchl, P. E., Projector Augmented-Wave Method. *Physical Review B* **1994,** *50* (24), 17953-17979.

(4) Grimme, S.; Antony, J.; Ehrlich, S.; Krieg, H., A Consistent and Accurateab Initioparametrization of Density Functional Dispersion Correction (Dft-D) for the 94 Elements H-Pu. *The Journal of Chemical Physics* **2010,** *132* (15).

(5) Halldin Stenlid, J.; Johansson, A. J.; Brinck, T., The Local Electron Attachment Energy and the Electrostatic Potential as Descriptors of Surface–Adsorbate Interactions. *Phys. Chem. Chem. Phys.* **2019,** *21* (31), 17001-17009.

(6) Momma, K.; Izumi, F. J. J. o. A. C., Vesta 3 for Three-Dimensional Visualization of Crystal, Volumetric and Morphology Data. **2011,** *44*, 1272-1276.

(7) Wang, V.; Xu, N.; Liu, J.-C.; Tang, G.; Geng, W.-T., Vaspkit: A User-Friendly Interface Facilitating High-Throughput Computing and Analysis Using Vasp Code. *Computer Physics Communications* **2021,** *267*.

(8) Maintz, S.; Deringer, V. L.; Tchougréeff, A. L.; Dronskowski, R., Lobster: A Tool to Extract Chemical Bonding from Plane-Wave Based Dft. *Journal of Computational Chemistry* **2016,** *37* (11), 1030-1035.

(9) <9-Nørskov-Et-Al-2004-Origin-of-the-Overpotential-for-Oxygen-Reduction-at-a-Fuel-Cell-Cathode.Pdf>.

(10) Wang, X. H.; Yuan, R.; Yin, S. B.; Hong, Q. L.; Zhai, Q. G.; Jiang, Y. C.; Chen, Y.; Li, S. N., Ultrathin Co0.5nis Nanosheets for Hydrazine Oxidation Assisted Nitrite Reduction. *Adv. Funct. Mater.* **2023,** *34* (8).

(11) Liu, Q.; Wen, G.; Zhao, D.; Xie, L.; Sun, S.; Zhang, L.; Luo, Y.; Ali Alshehri, A.; Hamdy, M. S.; Kong, Q.; Sun, X., Nitrite Reduction over Ag Nanoarray Electrocatalyst for Ammonia Synthesis. *Journal of Colloid and Interface Science* **2022,** *623*, 513-519.

(12) Murphy, E.; Liu, Y.; Matanovic, I.; Guo, S.; Tieu, P.; Huang, Y.; Ly, A.; Das, S.; Zenyuk, I.; Pan, X.; Spoerke, E.; Atanassov, P., Highly Durable and Selective Fe- and Mo-Based Atomically Dispersed Electrocatalysts for Nitrate Reduction to Ammonia Via Distinct and Synergized No2– Pathways. *ACS Catalysis* **2022,** *12* (11), 6651-6662.

(13) Wen, G.; Liang, J.; Liu, Q.; Li, T.; An, X.; Zhang, F.; Alshehri, A. A.; Alzahrani, K. A.; Luo, Y.; Kong, Q.; Sun, X., Ambient Ammonia Production Via Electrocatalytic Nitrite Reduction Catalyzed by a Cop Nanoarray. *Nano Res.* **2021,** *15* (2), 972-977.

(14) Liang, J.; Deng, B.; Liu, Q.; Wen, G.; Liu, Q.; Li, T.; Luo, Y.; Alshehri, A. A.; Alzahrani, K. A.; Ma, D.; Sun, X., High-Efficiency Electrochemical Nitrite Reduction to Ammonium Using a Cu3p Nanowire Array under Ambient Conditions. *Green Chem.* **2021,** *23* (15), 5487-5493.

(15) Wen, G.; Liang, J.; Zhang, L.; Li, T.; Liu, Q.; An, X.; Shi, X.; Liu, Y.; Gao, S.; Asiri, A. M.; Luo, Y.; Kong, Q.; Sun, X., Ni2p Nanosheet Array for High-Efficiency Electrohydrogenation of Nitrite to Ammonia at Ambient Conditions. *J. Colloid Interface Sci.* **2022,** *606*, 1055-1063.

(16) He, X.; Li, X.; Fan, X.; Li, J.; Zhao, D.; Zhang, L.; Sun, S.; Luo, Y.; Zheng, D.; Xie, L.; Asiri, A. M.; Liu, Q.; Sun, X., Ambient Electroreduction of Nitrite to Ammonia over Ni Nanoparticle Supported on Molasses-Derived Carbon Sheets. *ACS Applied Nano Materials* **2022,** *5* (10), 14246-14250.

(17) Wang, H.; Zhang, F.; Jin, M.; Zhao, D.; Fan, X.; Li, Z.; Luo, Y.; Zheng, D.; Li, T.; Wang, Y.; Ying, B.; Sun, S.; Liu, Q.; Liu, X.; Sun, X., V-Doped Tio2 Nanobelt Array for High-Efficiency Electrocatalytic Nitrite Reduction to Ammonia. *Materials Today Physics* **2023,** *30*.

(18) Liu, S.; Cui, L.; Yin, S.; Ren, H.; Wang, Z.; Xu, Y.; Li, X.; Wang, L.; Wang, H., Heterointerface-Triggered Electronic Structure Reformation: Pd/Cuo Nano-Olives Motivate Nitrite Electroreduction to Ammonia. *Appl. Catal. B-Environ.* **2022,** *319*.

(19) Ouyang, L.; Yue, L.; Liu, Q.; Liu, Q.; Li, Z.; Sun, S.; Luo, Y.; Ali Alshehri, A.; Hamdy, M. S.; Kong, Q.; Sun, X., Cu Nanoparticles Decorated Juncus-Derived Carbon for Efficient Electrocatalytic Nitrite-to-Ammonia Conversion. *Journal of Colloid and Interface Science* **2022,** *624*, 394-399.

(20) Li, X.; Li, Z.; Zhang, L.; Zhao, D.; Li, J.; Sun, S.; Xie, L.; Liu, Q.; Alshehri, A. A.; Luo, Y.; Liao, Y.; Kong, Q.; Sun, X., Ni Nanoparticle-Decorated Biomass Carbon for Efficient Electrocatalytic Nitrite Reduction to Ammonia. *Nanoscale* **2022,** *14* (36), 13073-13077.

(21) Qiu, H.; Chen, Q.; An, X.; Liu, Q.; Xie, L.; Zhang, J.; Yao, W.; Luo, Y.; Sun, S.; Kong, Q.; Chen, J.; Sun, X., Wo2 Nanoparticles with Oxygen Vacancies: A High-Efficiency Electrocatalyst for the Conversion of Nitrite to Ammonia. *J. Mater. Chem. A* **2022,** *10* (47), 24969-24974.

(22) Wang, J.; Liang, J.; Liu, P.; Yan, Z.; Cui, L.; Yue, L.; Zhang, L.; Ren, Y.; Li, T.; Luo, Y.; Liu, Q.; Zhao, X.-E.; Li, N.; Tang, B.; Liu, Y.; Gao, S.; Asiri, A. M.; Hao, H.; Gao, R.; Sun, X., Biomass Juncus Derived Carbon Decorated with Cobalt Nanoparticles Enables High-Efficiency Ammonia Electrosynthesis by Nitrite Reduction. *J. Mater. Chem. A* **2022,** *10* (6), 2842-2848.
